# Integrative single-cell and spatial transcriptome analysis reveals heterogeneity of human liver progenitor cells

**DOI:** 10.1097/HC9.0000000000000662

**Published:** 2025-02-26

**Authors:** Chuanjun Liu, Kai Wang, Junpu Mei, Ruizhen Zhao, Juan Shen, Wei Zhang, Liangyu Li, Bhaskar Roy, Xiaodong Fang

**Affiliations:** 1 College of Life Sciences, University of Chinese Academy of Sciences, Beijing, China; 2 BGI Research, Shenzhen, China; 3 Department of Hepatobiliary and Pancreatic Surgery and Minimally Invasive Surgery, Zhejiang Provincial People’s Hospital (Affiliated People’s Hospital), Hangzhou Medical College, Hangzhou, Zhejiang, China; 4 BGI Research, Sanya, China; 5 Hangzhou Institute of Medicine (HIM), Chinese Academy of Sciences, Hangzhou, Zhejiang, China

**Keywords:** cellular module, data integration, heterogeneity, liver progenitor cell, single cell, spatial transcriptome

## Abstract

**Background:**

Liver progenitor cells (LPCs) with bipotential differentiation capacities are essential for restoring liver homeostasis and hepatocyte population after damage. However, the low proportion and shared markers with epithelial cells make studying LPC heterogeneity difficult, especially in humans. To address this gap, we explored over 259,400 human liver single cells across 4 conditions (fetal, healthy, cirrhotic, and HCC-affected livers).

**Methods:**

Human liver tissue samples were analyzed using spatial transcriptomics sequencing technologies to describe the heterogeneity of LPCs. Liver tissue was characterized by LPC heterogeneity at single-cell resolution by employing cellular modules, differentiation trajectories, and gene co-expression patterns.

**Results:**

We annotated and identified 1 LPC cluster, 3 LPC subpopulations, and 4 distinct cellular modules, indicating the heterogeneity within LPC and the diversity between LPCs and epithelial cells. LPCs showed spatial colocalization with cholangiocytes and comprised a small proportion (2.95±1.91%) within the merged epithelial cells and LPC populations, exhibiting marked differences in marker expression patterns compared to those in mice. LPCs exhibited distinct cellular states in functional restoration, activation, proliferation, and cell transition. Additionally, the gene co-expression network of LPCs exhibited 3 unique modules, reflecting the distinct connectivity of genes encoding apolipoproteins and heat shock proteins in the gene co-expression network modules.

**Conclusions:**

Our study provides valuable insights into the multifaceted heterogeneity of human LPCs. Future studies focusing on spatial gene expression dynamics will contribute to our understanding of the spatial arrangement of liver regeneration.

## INTRODUCTION

The liver consists of hexagonal units called liver lobules, which comprise a diverse combination of epithelial and stromal cell lineages, contributing to its heterogeneity. Liver progenitor cells (LPCs) are bipotent, hepatoblast-like cells that can produce both hepatocytes and cholangiocytes. LPCs are pivotal in hepatic regeneration when the liver’s regenerative capacity is compromised in severe liver diseases. LPC-derived liver regeneration is critical in maintaining human liver homeostasis and development. Thus, promoting LPC-derived liver regeneration under various conditions could potentially benefit patients with severe liver disease.^1^ LPC heterogeneity is crucial in liver regeneration research, including variations within LPCs and their distinctions from epithelial cells (EPs), forming the essential microenvironment for LPC activation and differentiation in the human liver.^2^ Single-cell RNA sequencing (scRNA-seq) has improved the study of LPCs. However, it remains challenging to identify LPCs in small datasets due to their low proportion and shared markers with EPs.^3^,^4^ Consequently, few studies have investigated LPC heterogeneity at the single-cell level. LPCs are often grouped with cholangiocytes in scRNA-seq research, only a few studies have distinguished LPC clusters as bipotent cells.^4^,^5^ Besides these challenges in identifying LPCs through scRNA-seq, most conclusions of LPC-related research were based on rodent models and lack of LPC heterogeneity inspection. Due to the advantages of genetic manipulation, various dietary and toxin-induced models have been developed to mimic different aspects of LPCs.^1^,^6^ However, despite the usefulness of these models, there is significant cellular heterogeneity between humans and mice originating from physiology, behavior, pharmacokinetics, and genetics. It reveals the limitations of using mice as models for human liver diseases because of the heterogeneous cells.^7^ Even with substantial improvements, the reliability of animal studies in mimicking human LPCs remains a potential bias.^8^

Therefore, understanding the heterogeneity of human LPC populations and their functional attributes under various conditions is crucial for elucidating the intricate mechanisms underlying LPC-derived liver regeneration. Despite abundant scRNA-seq data revealing comprehensive transcriptional characteristics of cell types and regeneration, human LPC populations and their functional features in different conditions remain scarce. In the present study, we collected gene expression data of more than 259,400 single cells from 4 conditions of human fetal, healthy, cirrhotic, and HCC-affected livers. By identifying LPCs and elucidating their characteristics, we have revealed a wide range of heterogeneity within LPCs, and among EPs. This methodology offers valuable perspectives into human LPC heterogeneity under various conditions, facilitating the future exploration of condition-specific therapeutic strategies for liver diseases.

## METHODS

### Human liver tissue

Two human liver samples (STS1, STS2) were obtained from a deceased transplant-eligible donor. The collection was approved by the Institutional Review Board on the Ethics Committee of Beijing Genomics Institute (BGI-IRB-23075). Two pieces (50–200 mg each) were extracted from separate locations of the excised caudate lobe (segment 1). The liver blocks were quickly wiped dry, mixed with 4 °C optimal cutting temperature compound, embedded in a metal mold, frozen on dry ice, and stored at −80 °C. Detailed information is in the extended methods (Supplemental Method, http://links.lww.com/HC9/B944).

### Spatial transcriptome sequencing and data processing

We used a spatial enhanced resolution omics-sequencing (Stereo-seq) chip for in situ hybridization as previously described.^9^ The 10-μm thick tissue slice was transferred onto a −20 °C cryostat’s cold metal surface. After removing scattered tissue debris, the slice was positioned onto a prechilled −20 °C Stereo-seq chip. The experiment involved processing tissue with methanol fixation, fluorescent staining, and capturing nuclear ssDNA fluorescent images. The resulting cDNA underwent fragmentation, PCR amplification, and sequenced on an MGI DNBSEQ-Tx. Initially, raw reads were processed with the SAW pipeline (https://github.com/BGIResearch/SAW), encompassing mapping, merging, registration, counting, and tissue segmentation steps. The gene expression matrix (GEM), incorporating spatial coordinates, was further refined through image registration and tissue recognition. Stereocell was used to perform cell segmentation and generate single-cell GEM called cellbin.^10^ The cellbin annotation was conducted by R spacexr package v2.0.0.^11^ The spatial distance between each LPC and other cell types was calculated using the centroid coordinates of the 2 cells. The full description is in the extended methods (Supplemental Method, http://links.lww.com/HC9/B944).

### Single-cell GEMs collection and gene revision

GEMs from 42 individuals: fetal liver (FL, 6 individuals),^4^,^12^ healthy liver (HL, 14 individuals),^4^,^12^,^13^,^14^ cirrhotic liver (CL, 5 individuals),^14^ and HCC (17 individuals, 15 tumors [TL] and 8 adjacent tissues [TLA]),^4^,^15^ were collected from GEO with accession GSE134355,^12^ GSE136103,^14^ GSE146115,^15^ GSE115469,^13^ and GSE156337.^4^ Synonymous gene symbols were renamed to official identifiers using NCBI gene accession data.

### Data clustering and cell type assignment

To ensure accurate data clustering and cell type assignment, we implemented rigorous quality control measures. scRNA-seq GEMs quality control and subsequent analysis were performed using Seurat (v4.3.0) in R unless otherwise specified.^16^ Low-quality cells and genes were filtered based on 4 metrics: (1) the genes detected were above 200 and below 6000; (2) the total unique molecular identifier counts per cell were above 150; (3) the percentage of mitochondrial genes was below 50; (4) genes detected in at least 3 cells. DecontX was used to estimate ambient RNA, cells with a decontXcounts-calculated value exceeding 0.4 were excluded.^17^ DoubletFinder identified potential doublets, applying a 5% doublet rate threshold.^18^

We conducted a step-by-step process to integrate the divergent scRNA-seq data. Normalization and variance stabilization were performed using SCTransform.^19^ Subsequently, 3000 highly variable genes were selected, and the top 50 principal components were calculated using the RunPCA function. The Harmony algorithm corrected for donor, platform, and sorting biases, with theta values set to 4, 2, and 2.^20^ Clusters were determined using FindClusters with a resolution of 0.8, 1, 2, and 4, and the resolution of 2 was selected. After cell type annotation, we performed second-round clustering. Hepatocytes, cholangiocytes, and LPCs were integrated using canonical-correlation analysis with FindIntegrationAnchors and IntegrateData functions. HCC-specific clusters were identified based on their proportion across conditions and individuals. Gene counts were adjusted using PrepSCTFindMarkers, and cluster-specific markers were identified with FindAllMarkers; cell types were assigned based on classic markers,^21^,^22^,^23^,^24^,^25^,^26^ a progenitor subcluster (LPC_01_EPCAM) of 6754 cells annotated after second-round clustering. Detailed information is in the extended methods (Supplemental Method, http://links.lww.com/HC9/B944).

### Coembedding scRNA-seq and snRNA-seq data

To validate EPs from scRNA-seq datasets in capturing comprehensive cellular heterogeneity, we collected human single-nucleus RNA sequencing (snRNA-seq) data from HL, CL, and TL and assessed consistency between snRNA-seq and scRNA-seq subpopulations. Hepatocyte, cholangiocyte, and LPC subpopulations from HL (GSE185477),^27^ TL (GSE189175),^28^ and CL (GSE202379)^29^ were extracted. The function FindTransferAnchors and TransferData were performed to establish cell type correspondence between snRNA-seq and scRNA-seq. Detailed information is in the extended methods (Supplemental Method, http://links.lww.com/HC9/B944).

### Cellular modules of EPs and module score calculation

To determine LPC cellular patterns across 4 conditions, we calculated pairwise correlation values between cluster frequencies using the R cor function. These values were clustered to identify cellular modules (CMs) using the ward.D method and correlation distance. Differentially expressed genes (DEGs) among CMs were then identified, and the top 250 DEGs were compared across CMs. The gene ontology (GO), kyoto encyclopedia of genes and genomes, and gene set enrichment analysis enrichment were performed using clusterProfiler,^30^ with hallmark genes as reference for gene set enrichment analysis.^31^ To analyze the average expression of genes (*EPCAM*, *TACSTD2*, *FGFR2*, *TM4SF4*, *CLDN1*, *ANXA4*, *WWTR1*, *MYC*, *STMN1*, *PSMA4*, *SNRPB*, *ERH*, *NME1*, *TMEM14B*) associated with liver regeneration, we calculated the module score using AddModuleScore. The full description is in the extended methods Supplemental Method, http://links.lww.com/HC9/B944).

### Cell developmental trajectory of LPCs

We constructed LPC trajectories and performed pseudotime analysis with Monocle.^32^ The functions GetAssayData, new_cell_data_set, Embeddings, and learn_graph were sequentially executed for trajectory inference. The trajectory construction and pseudotime analysis among LPCs, cholangiocytes, and hepatocytes were performed using spaTrack (https://github.com/yzf072/spaTrack) under the “single-cell” model. The full description is in the extended methods (Supplemental Method, http://links.lww.com/HC9/B944).

### Co-expression network analysis of LPCs

Gene co-expression network (GCN) analysis was performed through hdWGCNA.^33^ The object of LPCs was built with SetupForWGCNA, wherein the option gene_select was set to fraction >5% cells. The TestSoftPowers function was used to assess soft power thresholds, and ConstructNetwork was executed to build the GCN. The full description is in the extended methods (Supplemental Method, http://links.lww.com/HC9/B944).

## RESULTS

### Integration of scRNA-seq data across 4 distinct conditions

**FIGURE 1:**
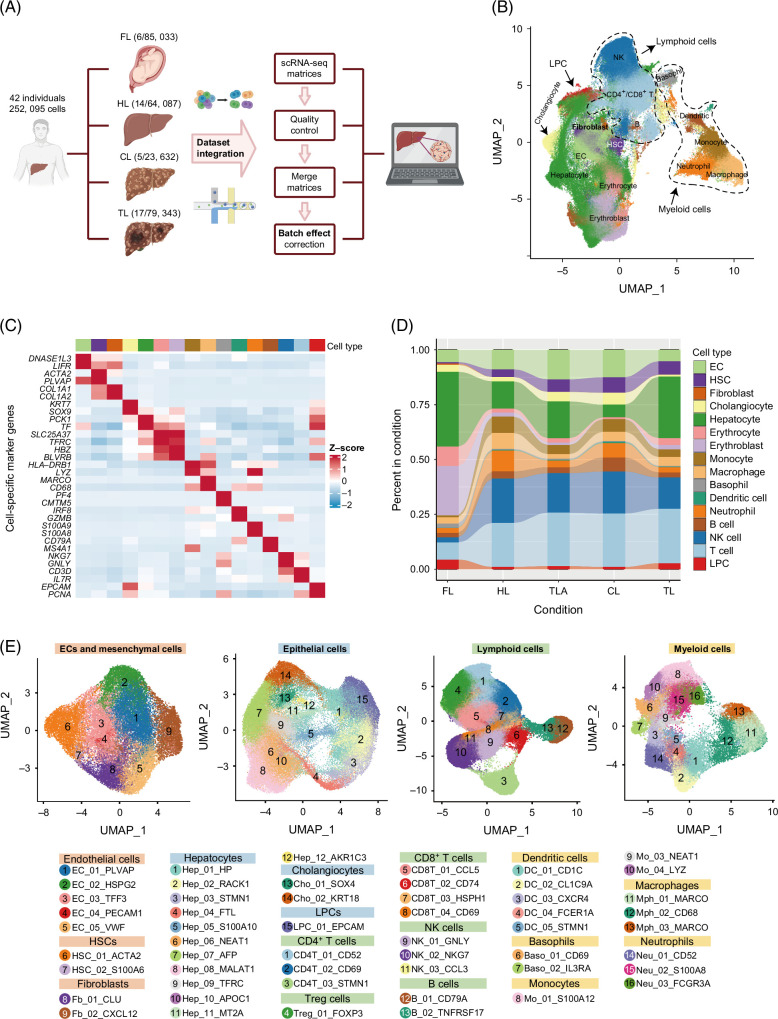
Integration of scRNA-seq and annotation of cells from the liver under 4 distinct conditions. (A) The schematic diagram illustrated the research workflow: scRNA-seq data from 4 conditions were collected and analyzed. (B) The UMAP clustering plot displayed 15 major cell types from 4 different conditions, including epithelial cells (hepatocytes, cholangiocytes), stromal cells (HSCs, fibroblasts), endothelial cells, erythrocytes, erythroblast, myeloid cells (basophils, neutrophils, dendritic cells, monocytes, and macrophages), and lymphocytes (NK cells, B cells, CD4^+^/CD8^+^ T cells). (C) Heatmap analysis used cell-specific marker genes to visualize the average gene expression across cell types. The z-score reflected the gene’s expression level deviation in different clusters from the overall average expression level. A positive z-score signified higher gene expression levels in the specific cluster compared to the average level, while a negative z-score indicated lower expression levels. (D) Sankey diagram illustrated the composition of liver cell types across 4 different conditions. (E) The UMAP plots showed subclusters of 4 major cell lineages: ECs and mesenchymal cells, EPs, lymphoid cells, and myeloid cells. Cluster color codes in the UMAP plots were consistent with cluster IDs in the legend. The rectangular boxes of legends with the same background color indicated that these cells belong to the same cell lineage. Abbreviations: CL, cirrhotic liver; FL, fetal liver; HL, healthy liver; LPC, liver progenitor cell; scRNA-seq, single-cell RNA sequencing; TL, HCC-affected liver of tumor; TLA, tumor-adjacent tissue; UMAP, uniform manifold approximation and projection.

To analyze the distribution of human LPCs across 4 conditions (FL, HL, CL, HCC [TL and TLA]) in a cohort of 42 individuals (Figure 1A, Supplemental Figures S1A and B, http://links.lww.com/HC9/B918, and Supplemental Table S1A, http://links.lww.com/HC9/B919), we carried out rigorous quality control measures, controlling mitochondrial gene percentage and removing doublets and contamination (Supplemental Figures S1E and F, http://links.lww.com/HC9/B918). Following Harmony integration (Supplemental Figure S1C, http://links.lww.com/HC9/B918), we observed a significant reduction in batch effects as evaluated by k-nearest-neighbor batch-effect test.^34^ Finally, more than 259,400 robust single cells were retained for further analysis. After excluding 4 clusters with <10 cells, we performed cell annotation through gene expression patterns and classic cell-specific markers (Figures 1B and C and Supplemental Table S1B, http://links.lww.com/HC9/B919). The annotated cell clusters included hepatocytes, cholangiocytes, endothelial cells, HSCs, fibroblasts, erythrocytes, erythroblasts, T cells (CD8^+^ and CD4^+^ T cells), NK cells, monocytes, macrophages, dendritic cells, and B cells. The uniform manifold approximation and projection (UMAP) plot was used to display cell types across 4 conditions (Figures 1B and D). Additionally, a second round of clustering was performed to characterize subpopulations of EPs, endothelial cells, mesenchymal cells, myeloid cells, and lymphoid cells (Figure 1E). In general, our extensive examination of a large number of single cells across 4 distinct conditions presents a valuable opportunity to depict the heterogeneity of LPCs.

### Putative bipotent LPCs

**FIGURE 2:**
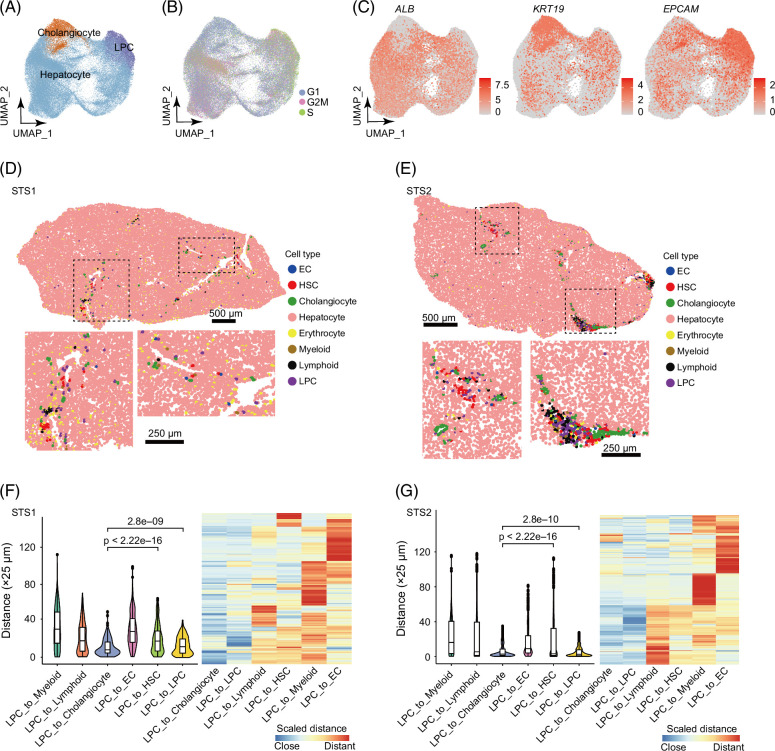
The distribution of LPCs and epithelial cells on UMAP and spatial transcriptome slices. (A) The UMAP plot displayed 3 major cell types of epithelial cells, including cholangiocytes, hepatocytes, and LPCs. (B) The effects of cell-cycle heterogeneity in epithelial cell clusters and cell-cycle phase scores were calculated based on canonical markers. (C) The dimensional reduction plot was according to the traditional markers of hepatocytes, cholangiocytes, and LPCs. (D, E) Cell types annotated from the spatial transcriptome of sample STS1 and STS2. (F, G) The distance between LPCs and other cell types on the spatial of samples STS1 and STS2 is calculated by the cellbin centroid coordinates of the LPCs and other cell types. Abbreviations: EC, endothelial cell; LPC, liver progenitor cell; UMAP, uniform manifold approximation and projection.

To recapitulate the heterogeneity of LPCs, we merged EPs (hepatocytes, cholangiocytes) and the classic progenitor marker *EPCAM* ^+^ populations for second-round clustering, and identified 15 subpopulations (Figures 1E and 2A). Cell-cycle scoring and regression were performed using GEMs to account for the effects of cell-cycle heterogeneity. The UMAP plot showed that the cell cycle did not significantly contribute to heterogeneity (Figure 2B). Although hepatocytes and cholangiocytes can differentiate into LPC following chronic liver injury, the cellular identity of the liver epithelial subset with LPC potential remains unclear.^35^ We compared an EP cluster LPC_01_EPCAM of *EPCAM* ^+^ and *PCNA* ^+^ with other clusters to identify the LPCs. The marker *EPCAM* encodes EP adhesion molecule (EpCAM), a progenitor marker found in a wide range of differentiated epithelia and is particularly prominent in tissue progenitors. LPCs highly express EpCAM at plasma membranes and decrease once morphogenesis is finished. The cluster LPC_01_EPCAM was enriched for *HNF1A* and *CDK1* (Supplemental Figure S2A, http://links.lww.com/HC9/B918), *HNF1A* encodes hepatocyte nuclear factor-1 alpha that linked with cell stemness,^36^ and *CDK1* is another LPC marker.^37^ Moreover, this cluster simultaneously expressed cholangiocyte markers (*SOX9* and *KRT7*) and hepatocyte markers (*PCK1* and *TF*) (Figure 1C), which were not present in other clusters. This hybrid cluster was consistent with LPC identified in former studies.^5^,^35^ The LPC_01_EPCAM subset exhibits a random expression pattern of the proliferation-associated markers *MKI67* and *PCNA*, highlighting the heterogeneity in transcriptional activity within this group that cannot be solely attributed to proliferation. Moreover, LPC_01_EPCAM expressed a group of proliferation and migration-related genes (Supplemental Table S2A, http://links.lww.com/HC9/B920) (*MYC*, *STMN1*, *PSMA4*, *SNRPB*, *ERH*, *NME1*, and *TMEM14B*),^5^ which are involved in cell proliferation, growth, and differentiation, suggesting that cells in this cluster played a crucial role in regulating cellular behavior. Hence, we defined this LPC_01_EPCAM population as LPCs, encompassing 6754 cells based on the comprehensive progenitor markers (Figures 2A and C and Supplemental Figure S2A, http://links.lww.com/HC9/B918).

### Spatial validation of LPCs in human liver tissue

To confirm the spatial distribution of LPCs in liver tissue, we performed spatial transcriptome (ST) experiments using Stereo-seq with 0.5 μm resolution.^9^ For Stereo-seq, each DNB corresponding to bin1, bin50 (50 bins × 50 bins) were 2500 bins, approximately equal to a hepatocyte with a diameter of 25–30 μm. Bin50 showed an average of 3719 (STS1), 4181 (STS2) mRNA molecules and 1074 (STS1), 1191 (STS2) genes (Supplemental Figures S3A and B, http://links.lww.com/HC9/B918). Cellbin exhibited 415 (STS1) and 451 (STS2) genes on average at single-cell resolution. The cellbin’s cell type on slides was determined using scRNA-seq data as a reference by robust cell type decomposition,^11^ which resulted in spatial annotation of LPC and 7 other major cell types (Figures 2D and E).

Spatial distances between LPCs and other cell types are vital for elucidating the cellular microenvironment of LPCs. We calculated the spatial distances between LPC and other cell types to investigate whether LPCs have a significant spatial colocalization relationship with cholangiocytes, HSCs, and other cells. We selected the distances to the 4 nearest cells of each cell type from LPCs to measure spatial colocalization. The results indicated that LPCs were spatially closer to cholangiocytes and themselves, whereas LPCs had greater spatial distances from HSCs and endothelial cells. Clustering the spatial distances between each LPC and other cell types revealed the same pattern (Figures 2F and G).

To investigate the LPC gene expression pattern between ST and scRNA-seq, we transformed the GEM of LPCs from STS1 and STS2, as well as scRNA-seq data, into pseudo-bulk RNA-seq gene expression data. We observed a significant correlation between gene expression of scRNA-seq and ST datasets STS1 (r=0.8283) and STS2 (r=0.8426). Additionally, there was a higher correlation between gene expression with STS1 and STS2 (r=0.9565) (Supplemental Figure S3C, http://links.lww.com/HC9/B918). We found that ST exhibited high expressions of *EPCAM*, *PCNA*, *HNF1A*, *CDK1*, *TET1*, *MET*, and *CD24* in LPC compared to other major cell types (Supplemental Figure S3D, http://links.lww.com/HC9/B918). These results indicate a significant similarity in gene expression patterns of LPCs between scRNA-seq and ST datasets.

### LPC heterogeneity within its population

**FIGURE 3:**
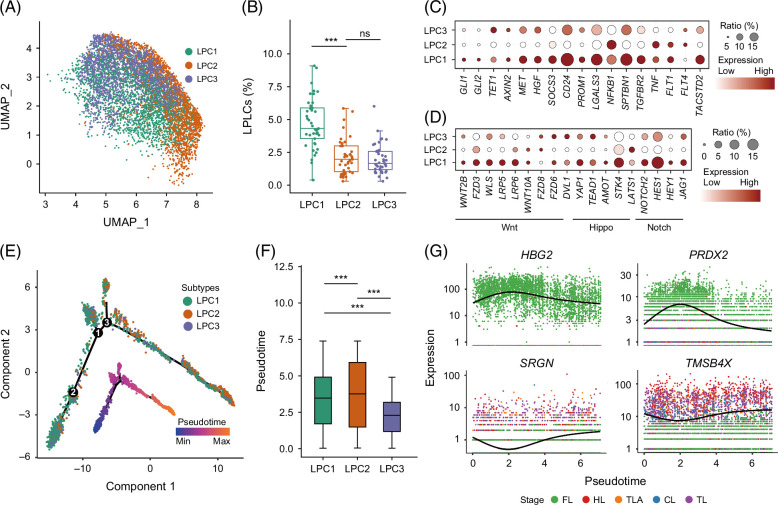
The subpopulation and trajectory of LPCs. (A) Clustering LPCs into 3 subtypes. (B) Box plots illustrated the proportions of LPC1–LPC3 in epithelial cells (hepatocytes and cholangiocytes) and LPCs, and significant differences were determined through the Wilcoxon testing. (C) The dot plot showed the expression and proportion of LPC markers in 3 subtypes. (D) Genes expression patterns of the LPC-related pathways, Wnt, Hippo, and Notch signaling. (E) In the diversity cell trajectory of LPC with Monocle, the colors of points represented 4 conditions, and the lines showed differentiation paths. Pseudotime on the trajectory graph, the color dark blue to light yellow indicates an increased pseudotime. (F) The boxplot illustrated the distribution of pseudotime across different conditions. (G) The expression characteristics of genes along the pseudotime and their distribution at different conditions. Colors indicated different liver conditions. Abbreviations: CL, cirrhotic liver; FL, fetal liver; HL, healthy liver; LPC, liver progenitor cell; TL, HCC-affected liver of tumor; TLA, tumor-adjacent tissue; UMAP, uniform manifold approximation and projection.

To characterize the LPC diverse heterogeneity within its population, we further re-clustered LPCs into 3 subpopulations, that is, LPC1 with *ALB* ^+^, LPC2 with *STMN1* ^+^, and LPC3 with *S100A6* ^+^. The genes expressed in a higher abundance and percentage of cells within LPC1–LPC3 were identified (Supplemental Table S2B, http://links.lww.com/HC9/B920), suggesting distinct cell states of heterogeneity within LPCs (Figure 3A). In LPC1, high expression of *APOA1*, which encodes apolipoprotein A1 (ApoA1), accelerates liver regeneration through regulating autophagy via AMPK–ULK1 pathway, ApoA1 deficiency impaired hepatocyte proliferation in vitro,^38^ suggesting that LPC1 was mainly involved in hepatocyte functional restoration during regeneration. In LPC2, high levels of *NPM1*, *ACTB*, *PTMA*, and *STMN1* are involved in centrosome duplication, protein chaperoning, and cell proliferation. *STMN1* expression alone can induce significant liver mass increase,^39^ suggesting LPC2 was prone to be activated. In LPC3, higher expression of *S100A6* and *S100A4*, members of the S100 protein family, which are known to modulate tissue regeneration and repair with substantially increased expression levels in EPs,^40^ suggesting LPC3’s involvement in cell transition during regeneration. Moreover, the critical genes in pathways that regulated LPC-derived regeneration, such as *WNT10A*, *FZD6*, *WLS*, and *LRP5* in Wnt signaling; *YAP1*, *TEAD1*, and *STK4* in Hippo signaling; *NOTCH2*, *HES1*, and *JAG1* in Notch signaling exhibited distinct expression fraction and abundance among LPC1–LPC3 (Figure 3D). In conclusion, LPC intrapopulation heterogeneity was characterized by distinct cell states, suggesting roles in hepatocyte restoration, cell activation, and cell transition.

LPC proportion in EPs represented heterogeneity within the LPC population. Our study assessed LPC proportions in merged epithelia and LPC across 4 conditions at 2.95±1.91% (n=42). LPC1 exhibited a significantly higher proportion than LPC2 and LPC3 (Figure 3B, Supplemental Figure S2B, http://links.lww.com/HC9/B918, and Supplemental Table S2C, http://links.lww.com/HC9/B920). These results aligned with previous research by Yamazaki et al^3^, who found similarly low LPC proportions in human liver with biliary atresia cirrhosis (2.9±0.4%, n=5) and control liver (0.9±0.4%, n=3). However, our results showed distinct proportions compared to mice under homeostasis and chronic injury states in 3,5-diethoxycarbonyl-1,4-dihydrocollidine-induced liver injury.^41^ Moreover, mice LPC markers, such as *Gli1* and *Axin2*,^1^,^42^ were seldom detected in human LPCs (Figure 3C). In contrast, human LPC markers, such as *CD24*, *NFKB1*, and *TACSTD2*, exhibited a conservative expression pattern with higher fraction and abundance. These findings indicated heterogeneous LPC populations between humans and mice in addition to the intrapopulation heterogeneity.

### Cell trajectory reveals the LPC heterogeneous state

LPC activation and differentiation result in cell heterogeneity as these processes undergo unique changes to take on specific roles within the liver. To further explore the heterogeneity of the LPC subpopulations during differentiation, the cell trajectory of LPCs was established with Monocle.^32^ The pseudotime distribution and cell state composition among LPC1–LPC3 displayed coordination, further confirming the validity of our trajectory analysis (Figure 3E). The 3 subpopulations exhibited a different pseudotime distribution when compared to the other 2 populations (Figure 3F). Additionally, LPC3 showed an earlier pseudotime. The trajectory and pseudotime of these LPC subpopulations suggested a heterogeneous state during LPC differentiation.

When splitting the pseudotime of each subpopulation according to the conditions, we found that the median pseudotime of FL, HL, and CL in LPC1 was relatively close, while in LPC2, there was a notable difference in median pseudotime among the conditions. Moreover, the CL in LPC2–LPC3 had an earlier median pseudotime (Supplemental Figure S2C, http://links.lww.com/HC9/B918). Furthermore, we found that the hepatocyte markers of FL decreased gradually along pseudotime, such as *HBG2* and *PRDX2*. On the other hand, the hepatic lipid metabolism regulation genes, such as *SRGN* and *TMSB4X*, mainly expressed in HL, CL, and TL, increased along pseudotime (Figure 3G), suggesting that LPCs represented heterogeneity within LPC populations and exhibited dissimilar cell states according to various conditions.

### Transcriptomic heterogeneity among EPs and LPCs across conditions

**FIGURE 4:**
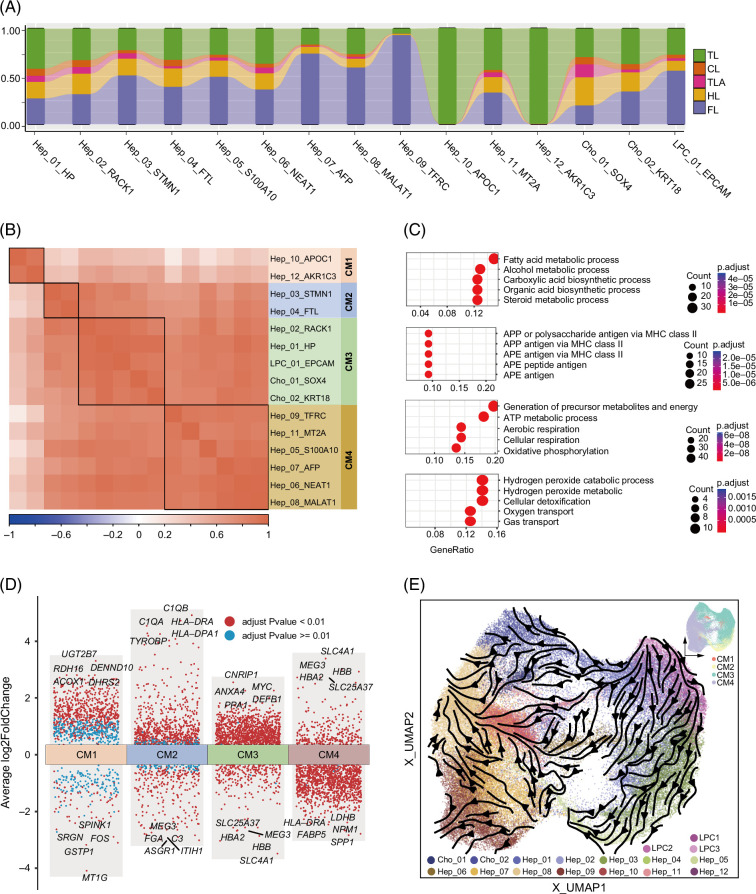
Heterogeneity and cellular module of epithelial cells. (A) The proportion of cells within each condition that constituted each subcluster. (B) The 4 cellular modules were based on correlations of cell clusters across 4 conditions. (C) The biological processes of gene ontology enrichment for CM1–CM4 markers were depicted in 4 figures, arranged in descending order from CM1 to CM4. (D) Differentially expressed genes of CM1–CM4 using average log2-fold change, adjusted *p* values <0.01 were highlighted in red, whereas adjusted *p* values ≥0.01 were displayed in blue. (E) The trajectory inference streamline plot of LPCs, cholangiocytes, and hepatocytes revealed that LPCs differentiate into cholangiocytes and hepatocytes along distinct transformative paths, the legend of the subclusters omitted gene names. The thumbnail in the top right shows the cell distribution of cellular modules. Abbreviations: APE, antigen processing and presentation of exogenous; APP, antigen processing and presentation; CL, cirrhotic liver; FL, fetal liver; HL, healthy liver; LPC, liver progenitor cell; TL, HCC-affected liver of tumor; TLA, tumor-adjacent tissue; UMAP, uniform manifold approximation and projection.

The heterogeneity of LPCs includes variations among the cells themselves and when compared to EPs. Understanding this heterogeneity is valuable for developing strategies to prompt LPC-derived liver regeneration. We identified more diverse cell populations in EPs due to the large cohort and 2 rounds clustering strategy. With markers *HP*, *RACK*, *STMN1*, *FTL*, *S100A10*, *NEAT1*, *AFP*, *MALAT1*, *TFRC*, *APOC1*, and *MT2A*, we annotated 11 hepatocyte subclusters. With markers *SOX4* and *KRT18*, we annotated 2 cholangiocyte subclusters (Figures 1E and 4A).

We compared these clusters’ origins to examine the heterogeneity and dynamics of cell subcluster composition across different conditions. The origin of the cell subcluster was associated with different conditions. Proportions of 6 subclusters of hepatocytes (from Hep_01_HP to Hep_06_NEAT1) remained relatively stable in HL but showed a decreasing trend in the cirrhotic stage. Moreover, 2 subclusters of hepatocytes (Hep_10_APOC1 and Hep_12_AKR1C3) mainly originated from TL. The composition of Cho_01_SOX4 and Cho_02_KRT18, 2 subtypes of cholangiocytes, exhibited opposite proportion trends between FL and HL; Cho_02_KRT18 showed a higher fraction in FL (Figure 4A and Supplemental Figure S2F, http://links.lww.com/HC9/B918). The fluctuating proportions reflected the extensive heterogeneity of hepatic EPs and LPCs under different conditions.

To confirm the heterogeneity of LPCs and EPs in scRNA-seq is comprehensive, we compared subclusters in snRNA-seq with those in scRNA-seq. In snRNA-seq, subtypes from 31,882 single nuclei under HL revealed that hepatocytes mainly matched 4 subclusters in scRNA-seq, while cholangiocytes mainly corresponded to Cho_02_KRT18. Furthermore, cholangiocytes exhibited LPC features in snRNA-seq, showing a higher proportion of LPC_01_EPCAM (Supplemental Figure S4A, http://links.lww.com/HC9/B918). Among 5297 single nuclei under CL, hepatocytes were mainly assigned to Hep_06_NEAT1 and Hep_08_MALAT1, with a low proportion of LPC_01_EPCAM in scRNA-seq. Among 23,385 single nuclei under TL, cells were assigned to 6 hepatocyte subclusters and 2 cholangiocyte subclusters in scRNA-seq (Supplemental Figures S4B and C, http://links.lww.com/HC9/B918). These findings highlighted the comprehensive representation of EPs and their heterogeneity in scRNA-seq data.

### CM analysis reveals 4 distinct constituent patterns

To investigate the constituent patterns of hepatic EPs and LPCs and underline their interactions, we explored the co-enrichment patterns of cells from these conditions. Four distinct CMs were identified through hierarchy clustering (Figure 4B). These CMs facilitated the stratification of LPCs and EPs into 4 corresponding cell constituent patterns. The properties of these patterns were based on 3 aspects: (1) cell clusters, (2) expression of marker genes associated with liver regeneration, and (3) markers of the subclusters. Among these CMs, CM3-enriched subcluster LPC_01_EPCAM, comprised 2 hepatocyte subclusters (Hep_01_HP, Hep_02_RACK1) and 2 cholangiocyte subclusters (Cho_01_SOX4, Cho_02_KRT18) (Figure 4B), exhibiting high expression of proliferation-related genes such as *MYC*, *ANXA4*, *DEFB1*, and *PPA1*. *MYC* regulates tissue regeneration, especially in the liver, which is crucial for liver regeneration. The enriched GO of ATP metabolic and cellular respiration (Figure 4C) reflected a metabolic state that promotes biosynthesis and mitosis, critical for tissue regeneration.^43^ The CM3 cell population was heterogeneous, consisting of subclusters inclined toward hepatocytes and cholangiocytes. This indicated that LPC_01_EPCAM had the potential to form bipotent capacity,^5^ thus indicating LPC_01_EPCAM has the potential for liver regeneration. By contrast, CM1, CM2, and CM4 contained only hepatocytes (Figures 4B and C). These different constituent patterns suggested that the cells in CM3 had a potential relation with LPC-driven liver regeneration.

To determine expression signatures among CMs, we identified DEGs as illustrated in the volcano plot (Figure 4D). We then identified the most abundantly expressed genes in each CM based on fold change and filtered the genes with an adjusted *p* value < 0.01. In comparison, CM3 showed the top 5 higher-level gene expressions of *CNRIP1*, *MYC*, *ANXA4*, *PPA1*, and *DEFB1* related to liver tissue regeneration and cell proliferation (Figure 4D). The discrete CM-specific gene signatures indicate their potential contribution to the maintenance of CM patterns and unique capacity in regeneration. The distribution of cells expressing DEGs varied significantly across the 4 CMs. We subsequently compared the proportion of cells expressing the top 250 DEGs (Figure 4D and Supplemental Table S2A, http://links.lww.com/HC9/B920) in each CM to those in the remaining 3 CMs (Supplemental Figure S2D, http://links.lww.com/HC9/B918). Each CM exhibited a higher proportion of cells than the other 3 CMs.

The trajectory inference of LPCs, cholangiocytes, and hepatocytes demonstrated LPCs’ potential to differentiate into both cholangiocytes and hepatocytes. Specifically, LPCs differentiated into Hep_01_HP, Hep_02_RACK1, and other hepatocyte populations, representing hepatic differentiation capacity. Additionally, LPCs differentiated into Cho_01_SOX4 and Cho_02_KRT18, indicating their potential to differentiate into cholangiocytes. The trajectory streamline plot revealed distinct transformative paths along which LPCs differentiate into cholangiocytes and hepatocytes (Figure 4E). The pseudotime distribution displayed on the UMAP plot showed LPCs with an early pseudotime, while hepatocytes and cholangiocytes exhibited late pseudotime. Moreover, *MYC*, *CNRIP1*, and *PPA1*, which are associated with liver regeneration and cell proliferation, exhibited higher expressions during early pseudotime (Supplemental Figure S2E, http://links.lww.com/HC9/B918), indicating their involvement in cell proliferation and tissue regeneration.

### The signatures of LPC among 4 conditions

**FIGURE 5:**
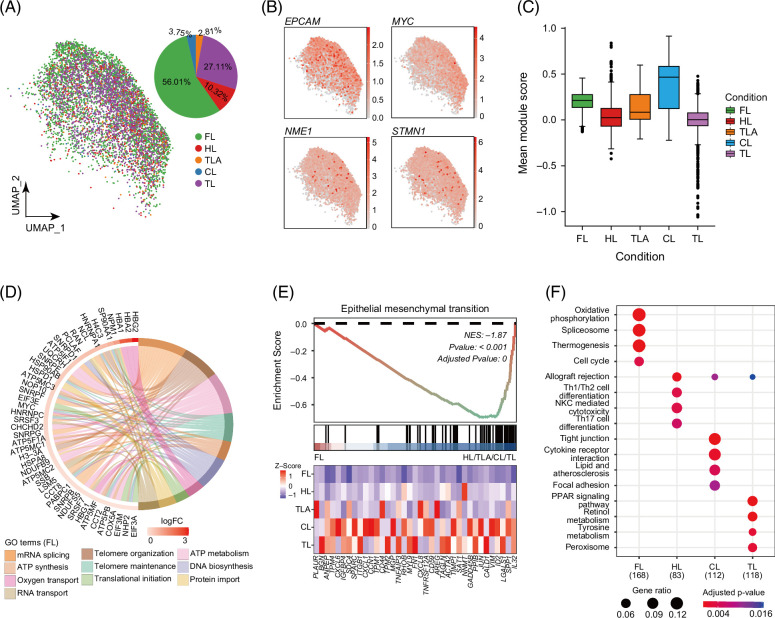
Signatures of LPCs among 4 conditions. (A) The UMAP analysis of LPCs from 4 distinct conditions constituent types were labeled in the UMAP map and pie chart. (B) The scale bar represented the normalized gene expression level of 4 liver progenitor makers. (C) Mean module score of LPC markers (*EPCAM, TACSTD2, FGFR2, TM4SF4, CLDN1, ANXA4, WWTR1, MYC, STMN1, PSMA4, SNRPB, ERH, NME1, TMEM14B*) that account for liver regeneration. (D) The circularly composited FL differentially expressed genes and their top 10 GO biological process. (E) Gene set heatmap and gene set enrichment analysis plot depicted the enrichment of epithelial–mesenchymal transition of FL. (F) The unique pathways of LPCs enriched in FL, HL, CL, and TL. Abbreviations: CL, cirrhotic liver; FL, fetal liver; GO, gene ontology; HL, healthy liver; LPC, liver progenitor cell; TL, HCC-affected liver of tumor; TLA, tumor-adjacent tissue; UMAP, uniform manifold approximation and projection.

To delineate the characteristics of LPCs across 4 conditions, we employed a UMAP plot to show LPCs and depicted LPC fluctuations using pie charts (Figure 5A). Notably, FL demonstrated a heightened proportion of progenitor cells. Feature plots were used to confirm the universal expression of *EPCAM*, *MYC*, *NME1*, and *STMN1* in LPCs (Figure 5B and Supplemental Figure S4D, http://links.lww.com/HC9/B918); these genes participate in various aspects of liver regeneration, including cell proliferation, differentiation, and migration. For instance, *EPCAM* increased significantly in the early LPC stage and gradually decreased with hepatocytic maturation.^44^ The mean module score of these genes showed different distributions, reflecting discrete signatures among 4 conditions (Figure 5C).

To explore LPC molecular alterations across these conditions, we compared GO enrichment. We found that the 4 conditions shared GO of reactive oxygen species (GO:0000302) and metabolic process of reactive oxygen species (GO:0072593), suggesting essential characteristics under differentiation conditions. Besides shared GO terms, LPCs at each condition possessed unique signatures of the GO biological process. LPCs of FL were characterized by telomere maintenance (GO:0000723) and DNA biosynthesis (GO:0071897) (Figure 5D). Telomere shortening in hepatocytes and lymphocytes can lead to viral replication in hepatitis and liver damage,^45^ suggesting that FL exhibited an advantage in maintaining telomere length during liver development. LPCs of HL were mainly related to protein secretion (GO:0050708) and EP proliferation (GO:0050673), which were consistent with the primary functions during liver regeneration (Supplemental Figure S5A, http://links.lww.com/HC9/B918). LPCs of CL-enriched wound healing (GO:0042060), and response to chemokine (GO:1990868) (Supplemental Figure S5B, http://links.lww.com/HC9/B918), chemokines are upregulated by macrophages and is vital in fibrotic niche formation.^46^ LPCs of TL-enriched intrinsic apoptotic signaling (GO:0097193) and response to hypoxia (GO:0001666) (Supplemental Figure S5C, http://links.lww.com/HC9/B918). The GO signature difference across these 4 conditions revealed a distinct response to stress and unique functions, suggesting LPC adaptations for differentiation or cell proliferation among varied conditions (Supplemental Figures S5D–G, http://links.lww.com/HC9/B918).

To further illustrate differences among these conditions, we analyzed events associated with epithelial–mesenchymal transition, a reversible alteration in EPs’ phenotype observed in wound healing, fibrosis, and metastasis. Gene set enrichment analysis and gene expression heatmaps confirmed epithelial–mesenchymal transition’s distinct gene expression pattern among FL and the other 3 conditions (Figure 5E). We performed the Kyoto Encyclopedia of Genes and Genome enrichment of DEGs among the conditions to determine LPC states associated with liver regeneration. The results showed unique pathways for each condition (Figure 5F). The oxidative phosphorylation, spliceosome, thermogenesis, and cell-cycle pathways were uniquely enriched in FL. While in CL, the uniquely enriched pathways were mainly immune cell differentiation and immune cell-mediated cytotoxicity. The distinct enrichment of terms and pathways among the conditions indicated varied regulation patterns among different conditions. The enrichment of these pathways in LPC emphasized their crucial role and underscored their significance in further exploring valuable strategies for liver regeneration.

### Distinct co-expression modules of LPCs

**FIGURE 6:**
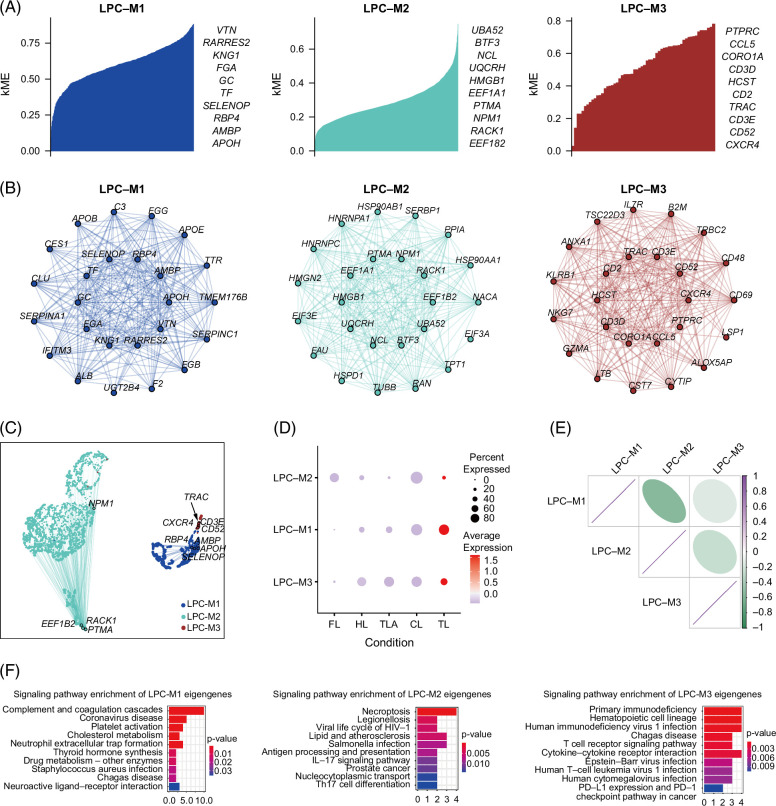
Co-expression network analysis based on LPC gene expression and signaling pathways of GCN eigengenes. (A) Genes in each module ranked by eigengene-based connectivity (kME) in LPC, where kME was calculated within a group comprising the 4 conditions and a total of 3 hdWGCNA modules were identified. (B) The module network of each module’s top 25 hub genes. Each plot depicted genes as nodes connected by edges denoting co-expression relationships. Node colors correspond to module assignments, with the top 10 hub genes centrally positioned and the remaining 15 arranged in the outer circle. (C) Co-expression networks visualization. The uniform manifold approximation and projection (UMAP) algorithm was applied to visualize the co-expression network data, and each point represented a single gene, each gene’s position in UMAP space based on its connectivity with the network’s hub genes. The size of each point corresponded to the gene’s kME value within its assigned module. The color of the points corresponded to the 3 different modules in A. (D) The dot plot showed harmonized module eigengenes (hMEs) by conditions. (E) Correlation heatmap of modules based on hub gene scores. (F) Signaling pathway enrichment of GCN top 25 eigengenes. Abbreviations: GCN, gene co-expression network; LPC, liver progenitor cell.

GCNs effectively reveal phenotypic variations in specific conditions by elucidating pairwise relationships between genes. A total of 3 GCN modules (LPC-M1/M2/M3) were identified; functionally important hub genes, which were interconnected nodes in each module, exhibited unique connective characteristics among the modules, suggesting that the hub genes possess a significant biological function within their modules during liver regeneration (Figure 6A).

In LPC-M1, apolipoprotein encoding genes (*APOB*, *APOE*, and *APOH*) had a universal connection with genes *CLU*, *GC*, *ALB*, and *TF*, which function in transport and maintain physiological balance. In LPC-M2, *HSP90AA1* and *HSP90AB1* exhibited co-expression with *EEF1A1* and *EEF1A2*, suggesting potential interactions between these translation elongation factors and heat shock proteins in coordinating cellular processes (Figure 6B). To explore the GCN gene connectivity within modules, we employed the UMAP algorithm to visualize the network by embedding the GCN into a low-dimension. The size of each dot was proportionate to the gene’s eigengene-based connectivity for its assigned module. LPC-M2 displayed a more intricate co-expression pattern, whereas LPC-M1 showcased a less complex GCN (Figure 6C). The expression percentages of harmonized module eigengenes indicated that FL and CL demonstrated a higher proportion of harmonized module eigengenes LPC-M2, while HL and CL exhibited a higher proportion in LPC-M3; TL showed higher abundance across all 3 modules (Figure 6D). Based on their hub gene scores, the correlation between each module revealed a negative relationship (Figure 6E). The underlying network modules with various co-expression patterns partly reflected characteristics of liver regeneration across conditions. This approach allowed us to comprehensively examine interactions and patterns within the entire network, enhancing our understanding of regulatory mechanisms underlying liver regeneration.

To investigate the functions of these GCNs in liver regeneration, we performed signaling enrichment on the top 25 eigengenes of each GCN (Figure 6F). The eigengenes (*HSP90AA1* and *HSP90AB1*) in LPC-M2 were enriched in the IL-17 signaling pathway, which has been shown to play a role in liver regeneration by promoting the expansion and differentiation of LPCs. Treatment with IL-17 has been found to promote the proliferation of LPCs.^47^ The eigengenes (*CCL5*, *CXCR4*, *LTB*, and *IL7R*) in LPC-M3 were enriched in cytokine–cytokine receptor interaction pathway, which is known to play a crucial role in coordinating cellular and molecular events during the early stages of liver regeneration.^1^ Signaling enrichment revealed that IL-17 and cytokine–receptor interaction pathways were important in association with liver regeneration in GCNs.

## DISCUSSION

Two obstacles have hindered research on LPCs. Firstly, the proportion of LPCs in liver tissue remains exceedingly low, even under conditions of chronic injury. Yamazaki and colleagues quantified the LPC proportions in human livers affected by biliary atresia–induced cirrhosis and in control livers using flow cytometry. Their findings indicated proportions of 2.9±0.4% (n=5) and 0.9±0.4% (n=3), respectively.^3^ Meanwhile, Pu and colleagues employed a fumarylacetoacetate hydrolase gene knockout mouse model, which triggers hepatocyte senescence during liver regeneration. Their study revealed that LPCs constitute 3% of the *EPCAM* ^+^ EP clusters.^35^ This scarcity poses challenges in identifying LPCs within small datasets derived from scRNA-seq. Secondly, the overlapping cellular markers between EPs and LPCs complicate accurately identifying LPCs.^4^ Our study integrated 5 scRNA-seq datasets from 4 distinct conditions, encompassing over 259,400 single cells. We pinpointed a specific cluster of bipotent progenitors by implementing a 2-round clustering approach. The expansive scale of our dataset serves as a substantial foundation for the comprehensive investigation of LPCs.

Studying human LPCs’ heterogeneity is crucial for understanding liver regeneration, which contributes to maintaining liver homeostasis and recovery following injury. The previous studies have primarily relied on rodent models subjected to specific conditions. Nevertheless, despite the utility of rodent models, notable cellular heterogeneity exists between humans and mice due to differences in physiology, behavior, pharmacokinetics, and genetics. In this research, we analyzed gene expression profiles from over 259,400 single cells across distinct conditions, including fetal, healthy, cirrhotic, and HCC-affected livers, unveiling a range of heterogeneous LPCs. Our study identified LPCs by progenitor traditional marker *EPCAM* and genes associated with hepatic stem cells,^5^,^48^ such as *HNF1A* and *CDK1*, aligning our research with previous work on liver development and regeneration. The identification and characterization of LPCs and their distinctive CMs in liver-related regeneration revealed heterogeneity among EPs. This comprehensive approach offers valuable insights into LPCs under different liver conditions and holds promise for developing condition-specific strategies to treat liver diseases.

LPCs displayed significant heterogeneity within their population, forming 3 distinct subpopulations (LPC1–LPC3). LPC1 suggested its involvement in hepatocyte restoration, LPC2 indicated a propensity for activation and proliferation, while LPC3 likely played a role in facilitating cell transition during regeneration. Moreover, critical genes in pathways regulating LPC-derived liver regeneration, including Wnt, Hippo, and Notch signaling, exhibited distinct expression patterns across LPC1–LPC3. The proportion of LPCs within EPs further underscored LPC heterogeneity, with LPC1 showing a significantly higher proportion compared to LPC2 and LPC3. Human LPC markers, such as *CD24*, *NFKB1*, and *TACSTD2*, exhibited a conservative expression pattern, further highlighting heterogeneous LPC populations between humans and mice and intrapopulation heterogeneity. In this study, the inclusion of the CL and the TL provided enriched conditions for observing LPC heterogeneity. Given the conflicting findings regarding the role of LPC within cirrhosis and HCC,^1^ our focus was on understanding the heterogeneity of LPC across different conditions. We refrained from making inferences regarding whether LPC promotes liver fibrosis or the development of liver cancer under the cirrhosis and HCC conditions.

Despite characterizing LPCs across conditions using scRNA-seq data, our study has certain constraints in exploring liver lobule zonation and spatial gene expression patterns. The spatial organization of cells and gene expression patterns is vital in liver regeneration. In our study, we found a close spatial proximity between LPCs and cholangiocytes, this spatial distribution may indicate a cooperative role of LPCs and cholangiocytes in the regenerative process. Furthermore, understanding the spatial organization of LPCs can provide insights into their niche and microenvironment in the liver, which may influence their behavior and function during regeneration. Future research efforts should address these gaps by integrating spatial transcriptomics techniques to unveil spatial gene expression, cell–cell interactions, and zonation in liver regeneration. In the future, studies concentrating on spatial gene expression dynamics will offer helpful insights into the spatial organization of liver regeneration. Ultimately, a more profound understanding of these aspects could guide future policies and practices, particularly in personalized medicine approaches for liver disease therapies.

## Supplementary Material

## Data Availability

The integrated scRNA-seq matrices, supplemental tables, and metadata generated in the present study are available in the figshare repository (https://figshare.com/s/8cfa63bab6af19b8d7ce). The original scRNA-seq matrices are publicly available at GEO under accession numbers GSE115469,^13^ GSE134355,^12^ GSE136103,^14^ GSE146115,^15^ and GSE156337.^4^ Scripts for data analysis and figures can be accessed via the above-mentioned link. Chuanjun Liu: formal analysis, visualization, and writing. Kai Wang: investigation and formal analysis. Junpu Mei: methodology and visualization. Ruizhen Zhao, Juan Shen, Wei Zhang, and Liangyu Li: formal analysis, conceptualization, and investigation. Bhaskar Roy: writing and editing. Xiaodong Fang: funding acquisition, supervision, and investigation.
