# Supplementary material for: Integrative single-cell and spatial transcriptome analysis reveals heterogeneity of human liver progenitor cells
Source: Hepatol Commun. 2025 Feb 26;9(3):e0662. doi: 10.1097/HC9.0000000000000662 (PMC11868439; doi:10.1097/HC9.0000000000000662)

Supplementary_Figure1


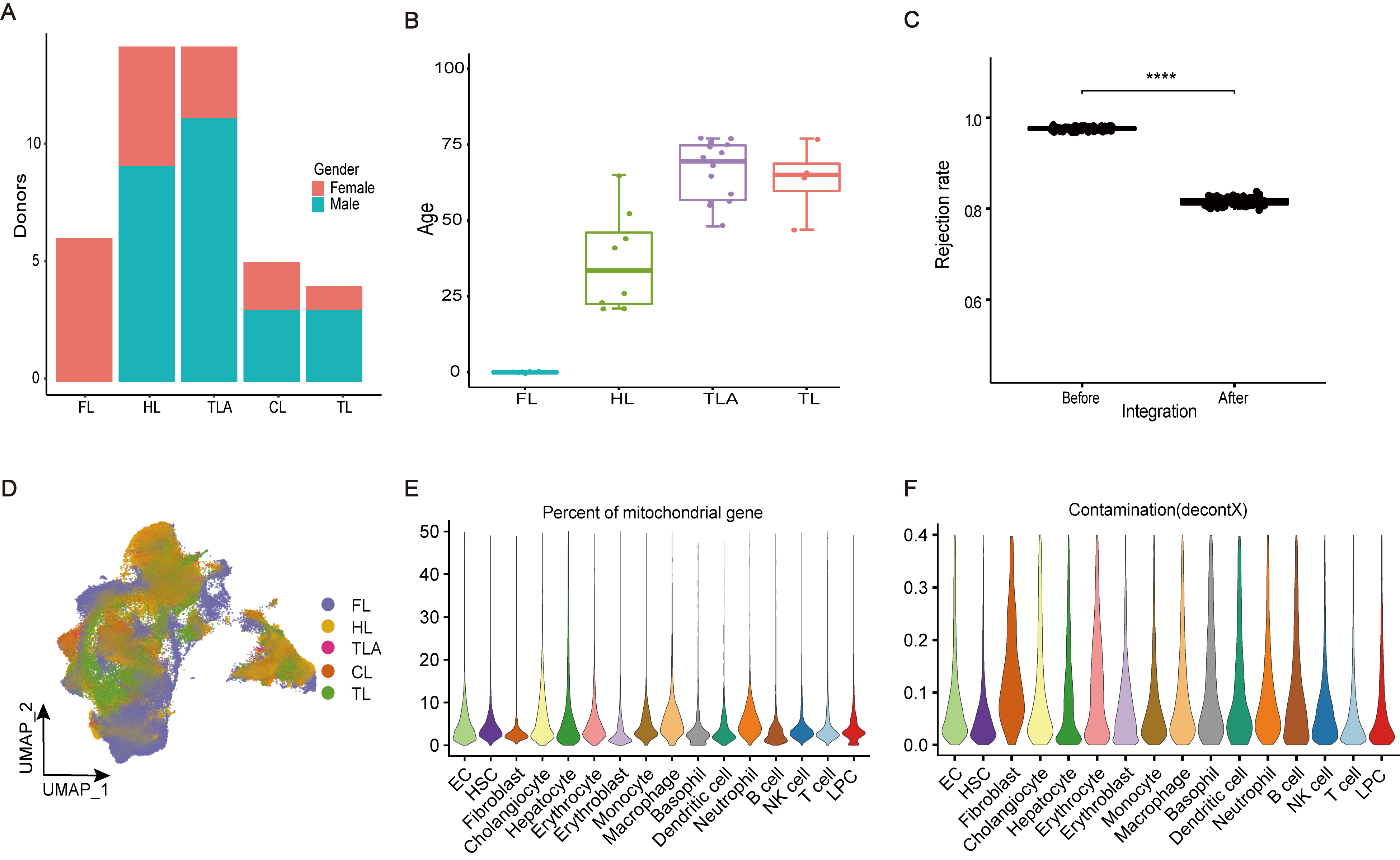


Supplementary_Figure 2


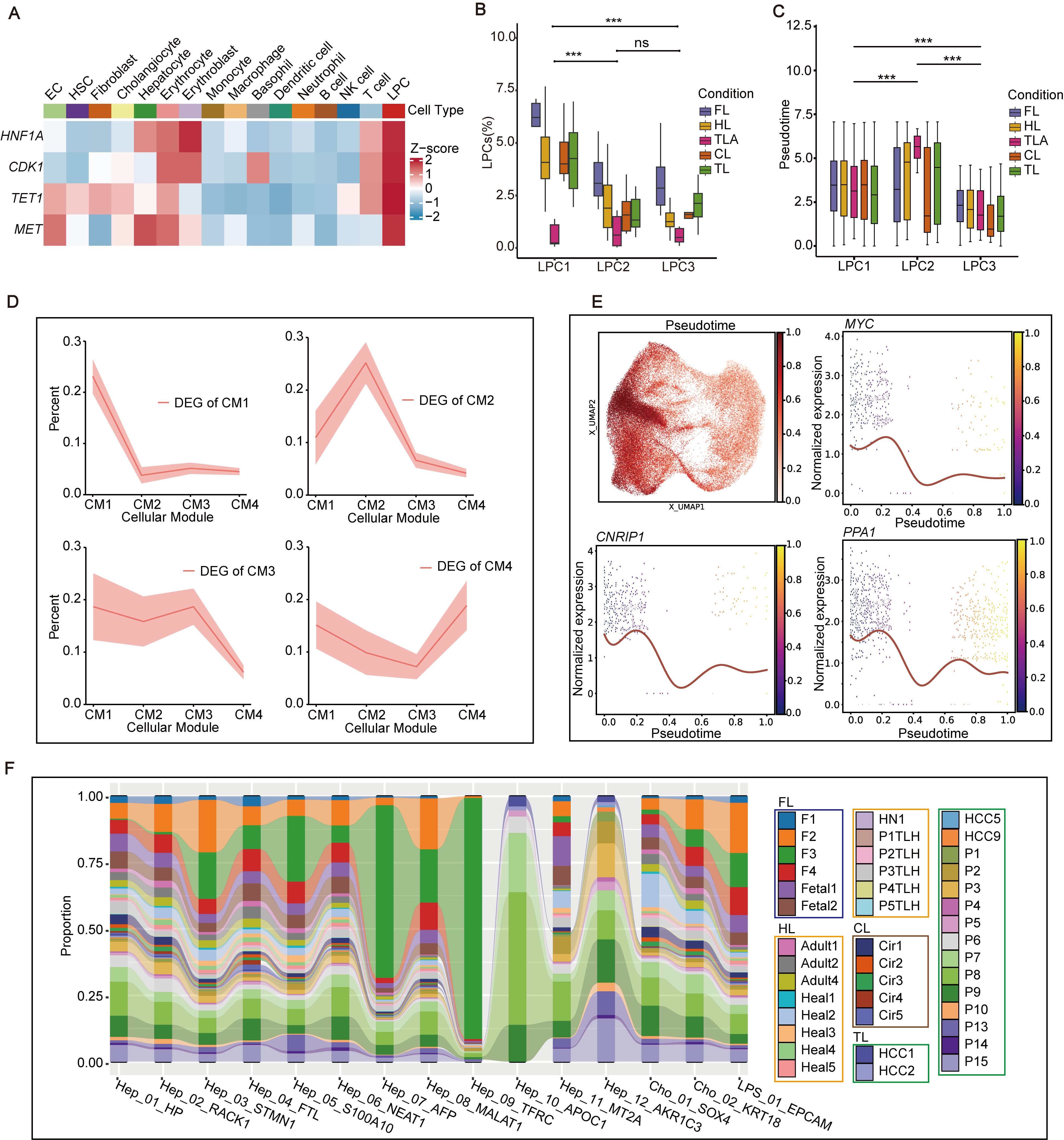


Supplementary_Figure 3


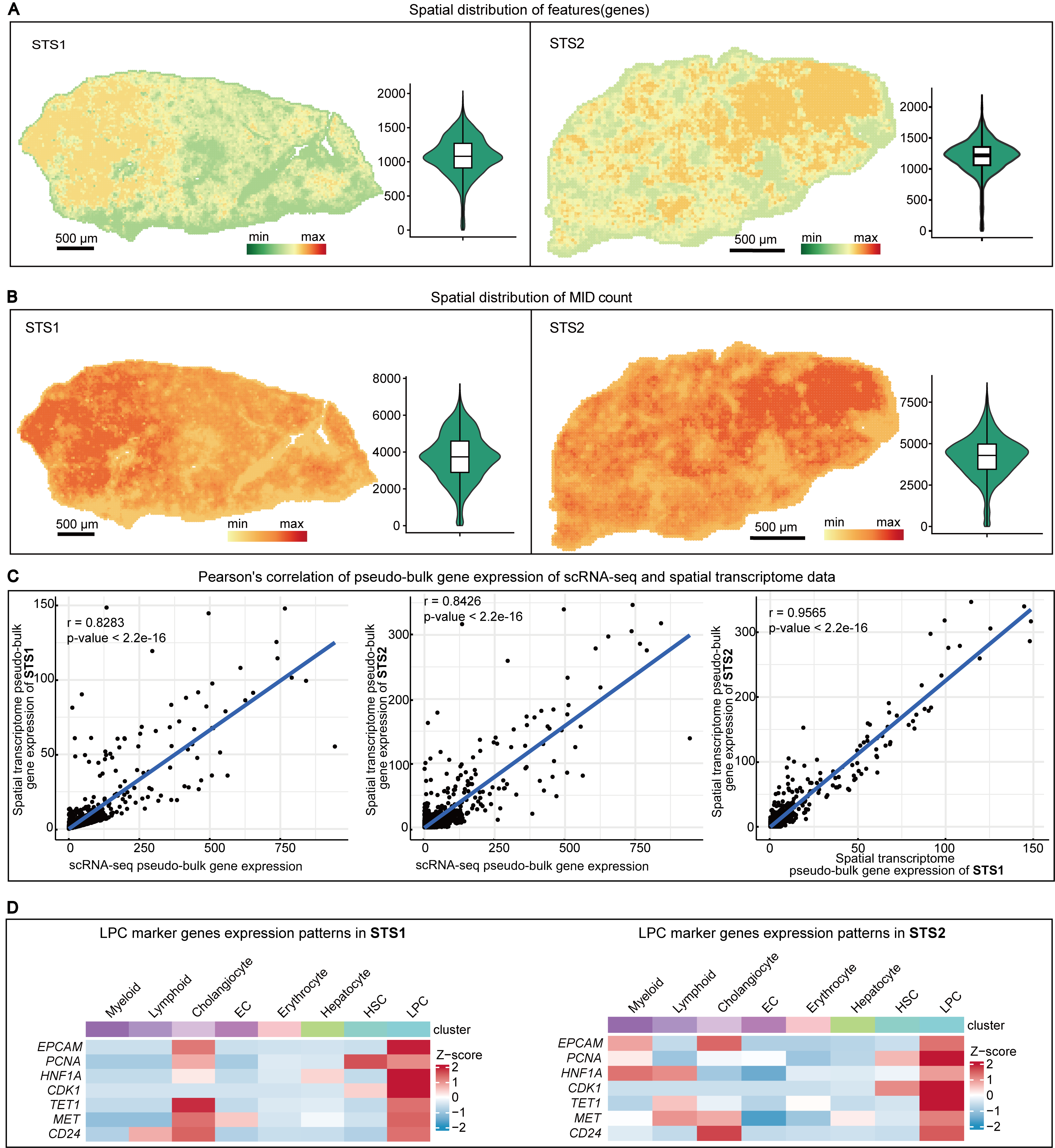


Supplementary_Figure 4


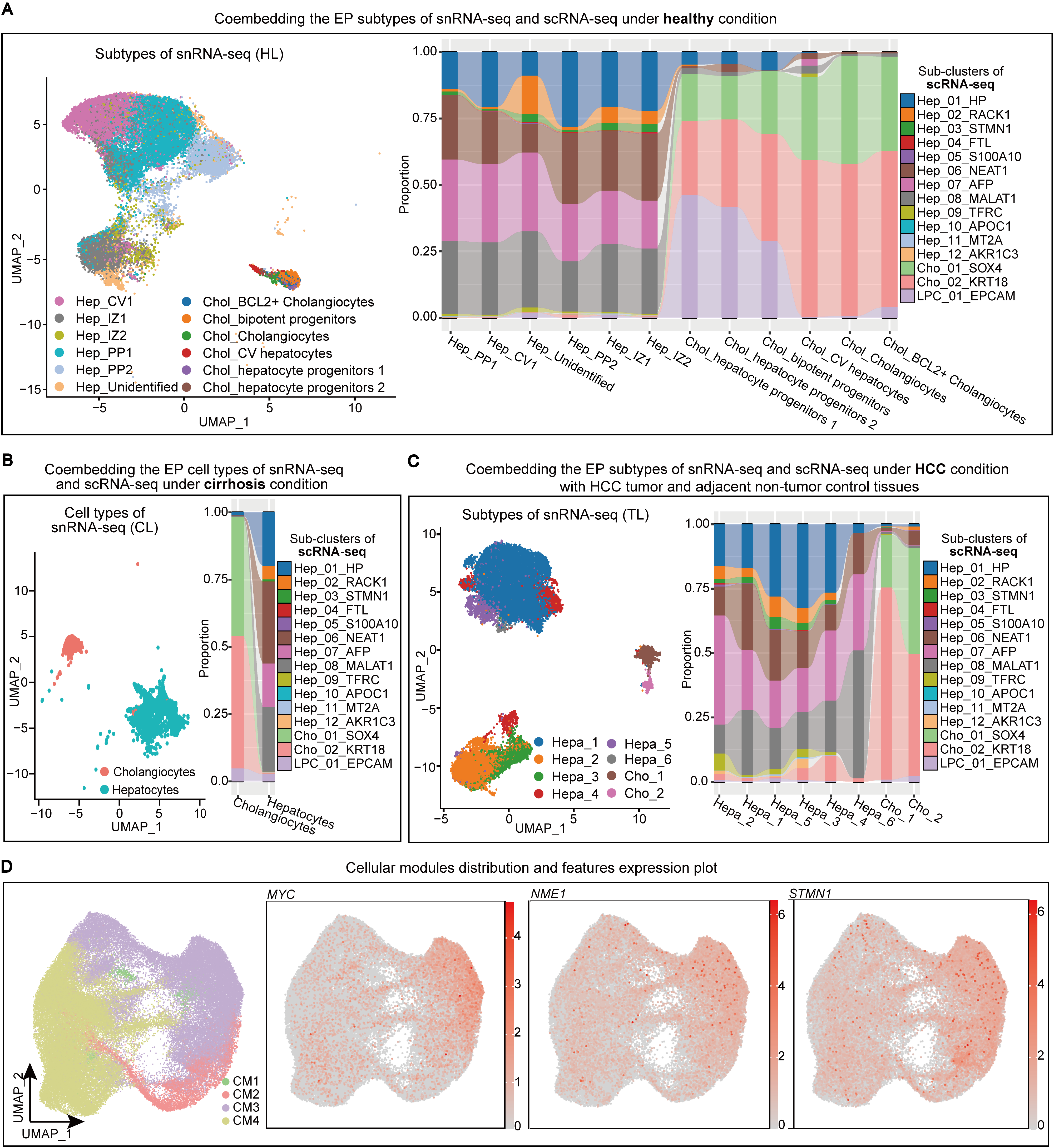


Supplementary_Figure 5


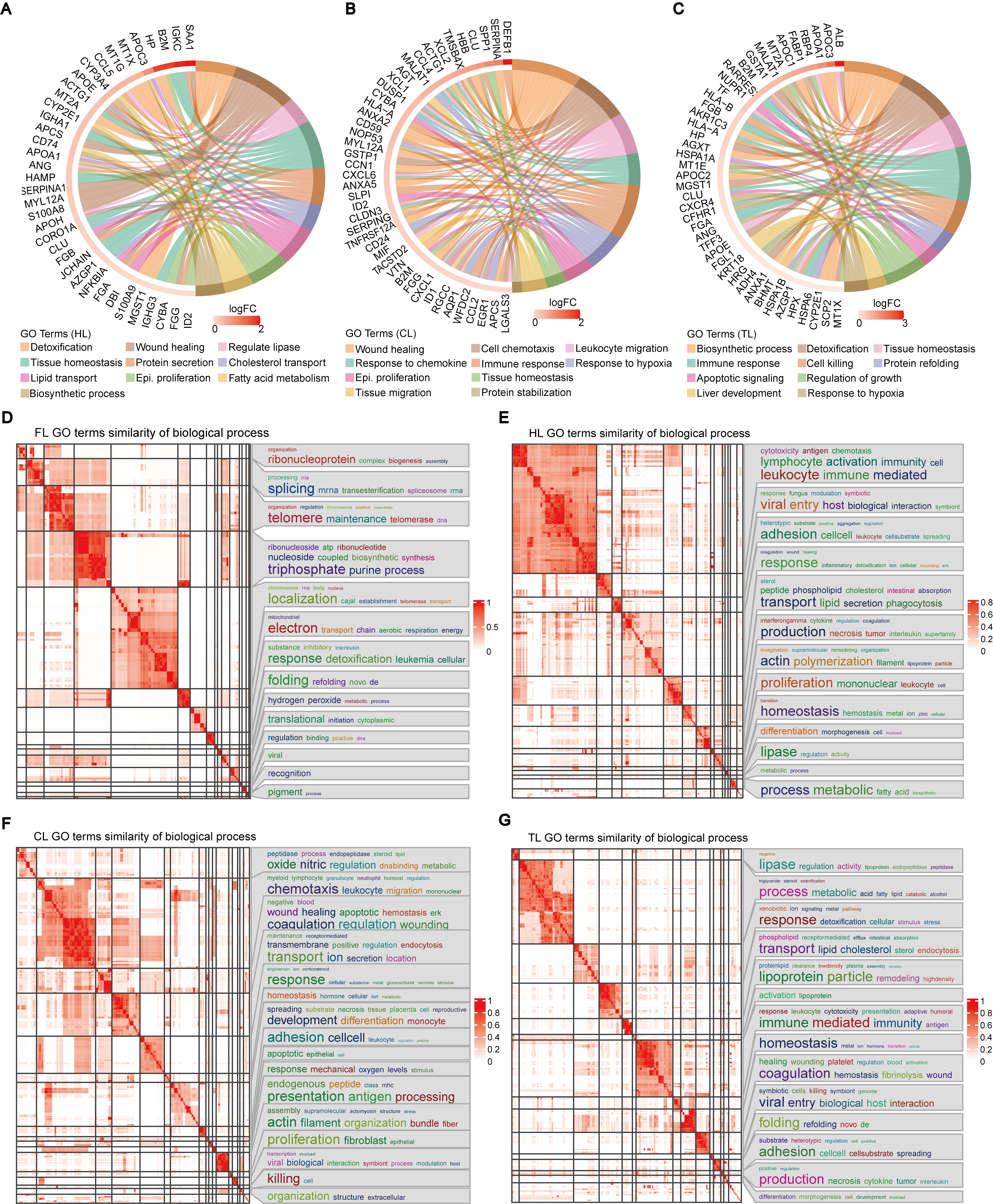

Supplement: Supplementary file 1 [file hc9-9-e0662-s001.docx]
