# Supplementary material for: Integrative single-cell and spatial transcriptome analysis reveals heterogeneity of human liver progenitor cells
Source: Hepatol Commun. 2025 Feb 26;9(3):e0662. doi: 10.1097/HC9.0000000000000662 (PMC11868439; doi:10.1097/HC9.0000000000000662)
Supplement: Supplementary file 4 [file hc9-9-e0662-s004.pdf]

Supplemental Digital Content

Integrative single-cell and spatial transcriptome analysis reveals heterogeneity of human liver progenitor cells

Chuanjun Liu, Kai Wang, Junpu Mei, Ruizhen Zhao, Juan Shen, Wei Zhang, Liangyu Li, Bhaskar Roy, Xiaodong Fang

List of Supplementary Figures and Methodological Details

SUPPLEMENTARY FIGURES 2

Supplementary Figure 1. The donors comprised the cohort and the data quality control .. 2

Supplementary Figure 2. Signatures of LPC within its group and among epithelial cells ... 3

Supplementary Figure 3. The spatial distribution of features and MIDs 4

Supplementary Figure 4. Coembedding two platforms' data and cellular module features 5

Supplementary Figure 5. Enrichment of Biological Processes in different conditions 6

EXTENDED METHODS 7

Human liver tissue 7

Spatial transcriptome sequencing experiment 7

Stereo-seq data processing 8

Single-cell gene expression matrices collection and gene revision 9

scRNA-seq matrices quality control 9

Data integration and clustering 9

Marker identification and cell type assignment 10

Coembedding the scRNA-seq and single nucleus RNA sequencing data 10

Cellular modules of epithelial cells 11

Module score calculation for regeneration-related genes 11

Cell developmental trajectory of LPCs 12

Co-expression network analysis of LPCs 12

Bibliography 13

SUPPLEMENTARY FIGURES

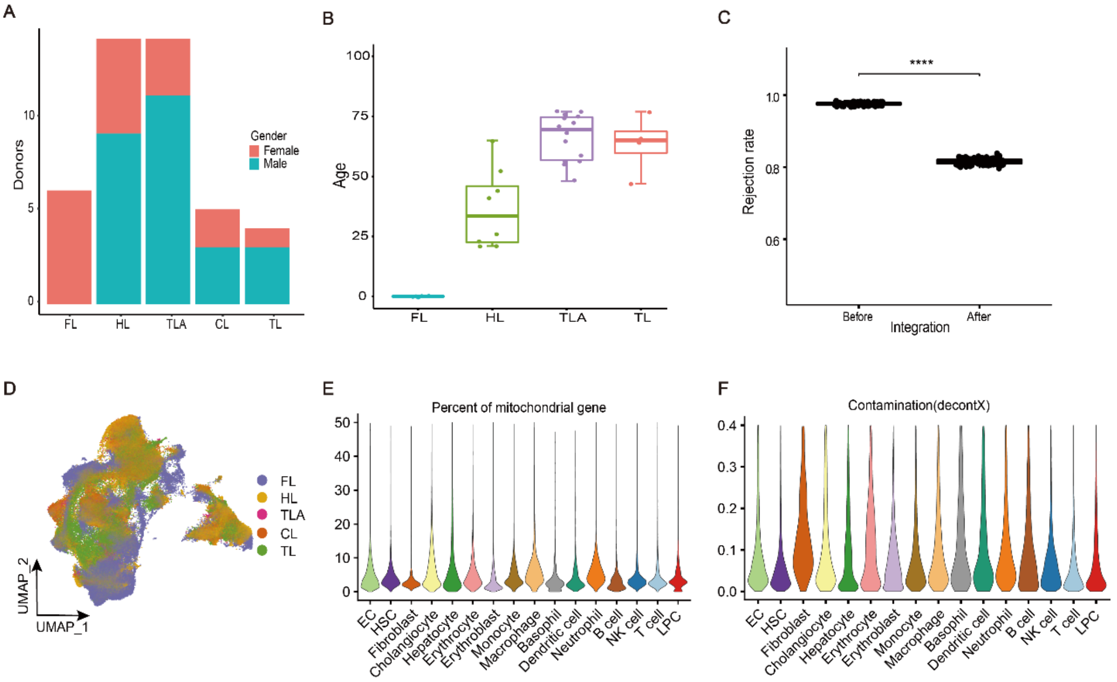

Supplementary Figure 1. The donors comprised the cohort and the data quality control.

(A) The histogram showed the composition of donor gender in our cohort and (B) of donor age. (C) The rejection rates before and after integration, which represented the level of batch effects, were assessed by R package kBET; batch effects were significantly reduced after integration. (D) UMAP plots showed the distribution of liver states. (E) Percentage of mitochondrial genes among the different cell types. (F) The contaminations score was calculated using the R package DecontX.

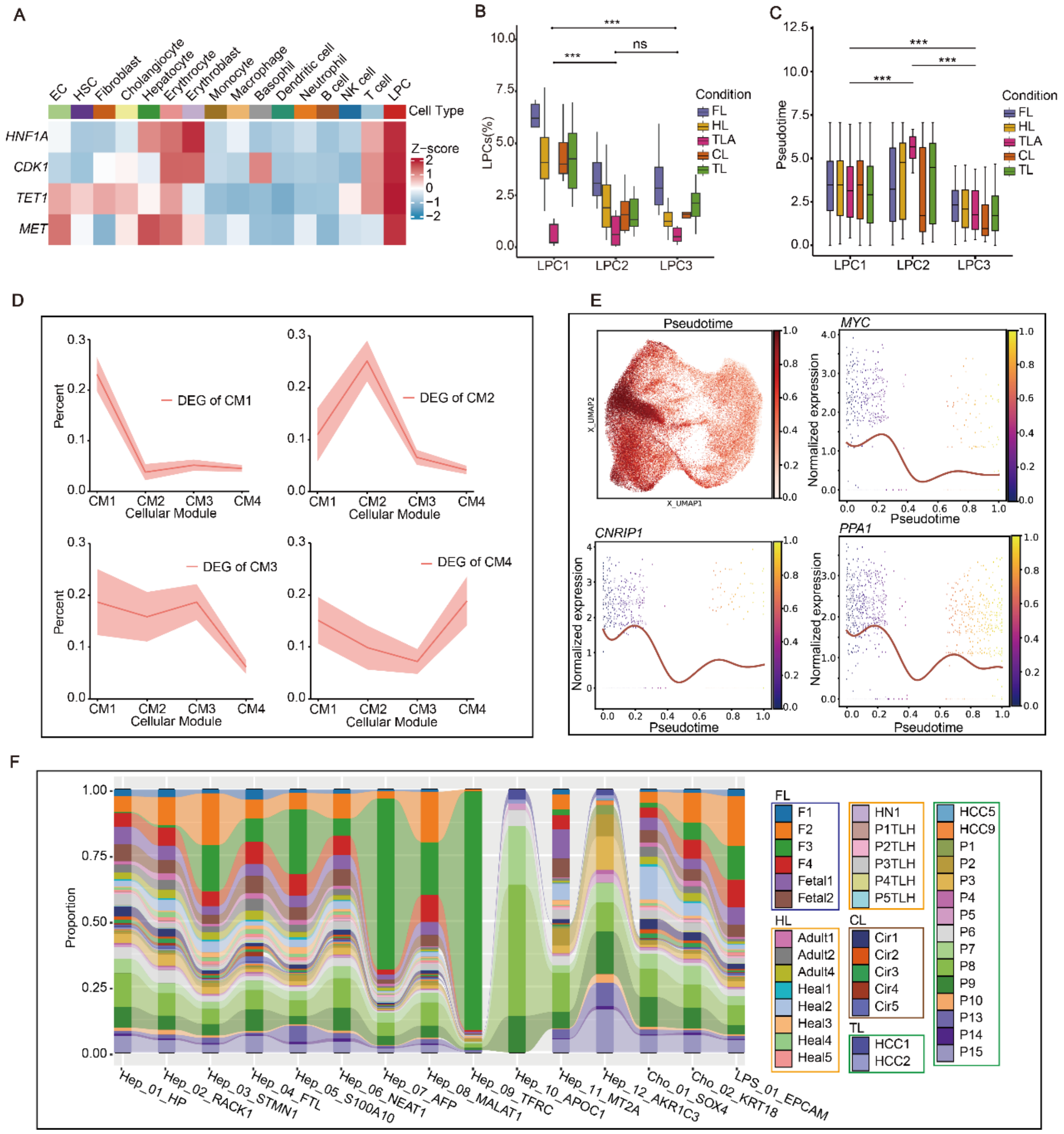

Supplementary Figure 2. Signatures of LPC within its group and among epithelial cells.

(A) The heatmap of LPC markers. (B) Box plots illustrating the proportions of LPC1-LPC3 in epithelial cells across four conditions, significant differences calculated with wilcoxon-test. (C) The figure showed the pseudotime of LPC1-LPC3 under different conditions. (D) The percentage of cells expressing the DEGs in CM1-CM4, a 95% confidence interval (shadow), was calculated for each cellular module (solid line). (E) The pseudotime distribution on the UMAP plot showed that LPCs exhibited an early pseudotime, while hepatocytes and cholangiocytes exhibited a late pseudotime. The higher-level expressions of *MYC*, *CNRIP1*, and *PPA1*, which are related to liver tissue

regeneration and cell proliferation, were observed during the early pseudotime. (F) The proportion of cells within individuals that constituted each sub-cluster.

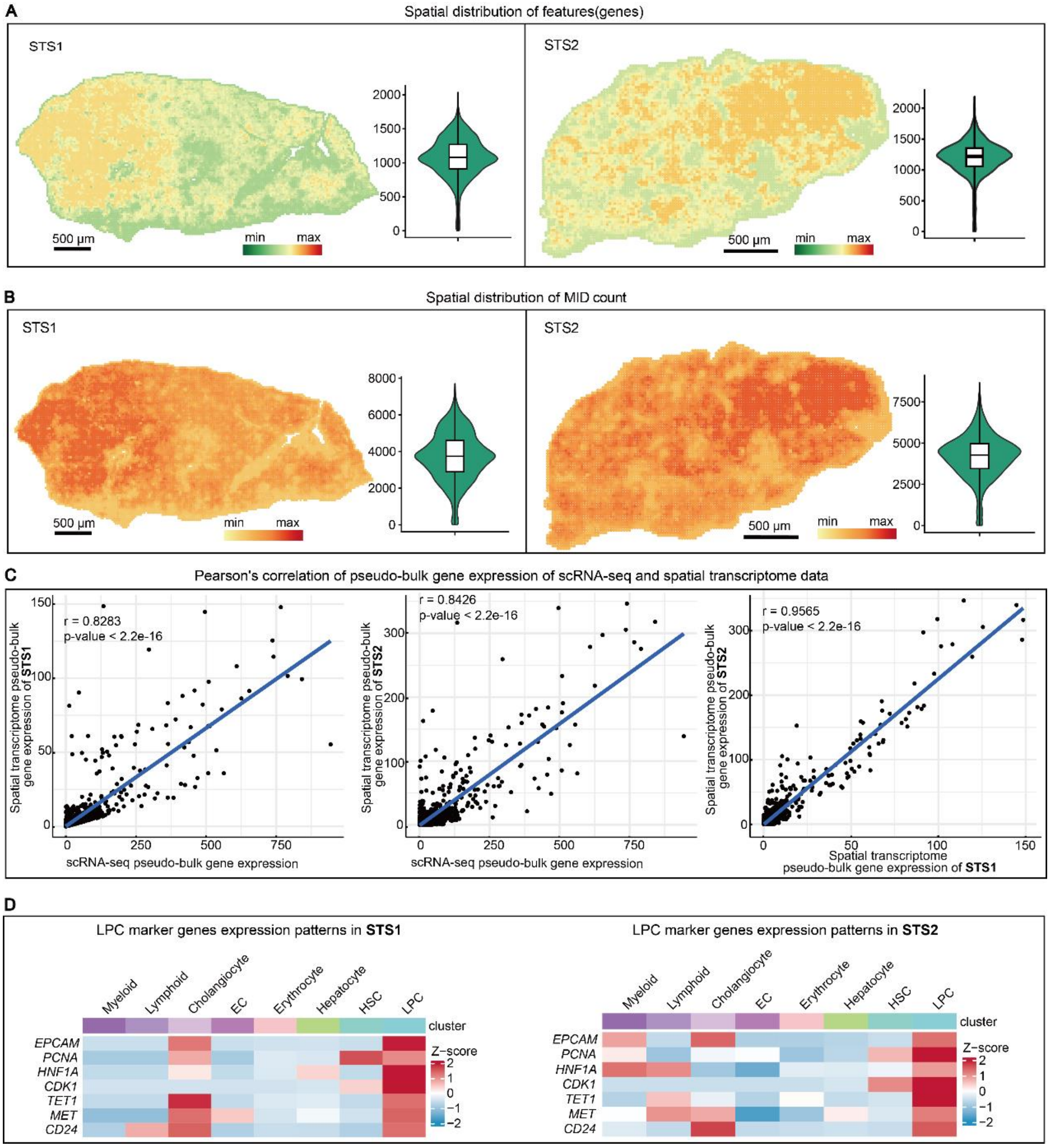

Supplementary Figure 3. The spatial distribution of features and MIDs.

(A) Features number of STS1 and STS2 on spatial slices, showed features(genes) number of bin50 square area. (B) The MID counts of STS1 and STS2 on spatial slices, calculated under bin50 square area. (C) The correlation of pseudo-bulk gene expression between scRNA-seq and spatial transcriptome datasets (STS1 and STS2).

(D) Gene expression level of *EPCAM*, *PCNA*, *HNF1A*, *CDK1*, *TET1*, *MET*, and *CD24* in STS1 and STS2.

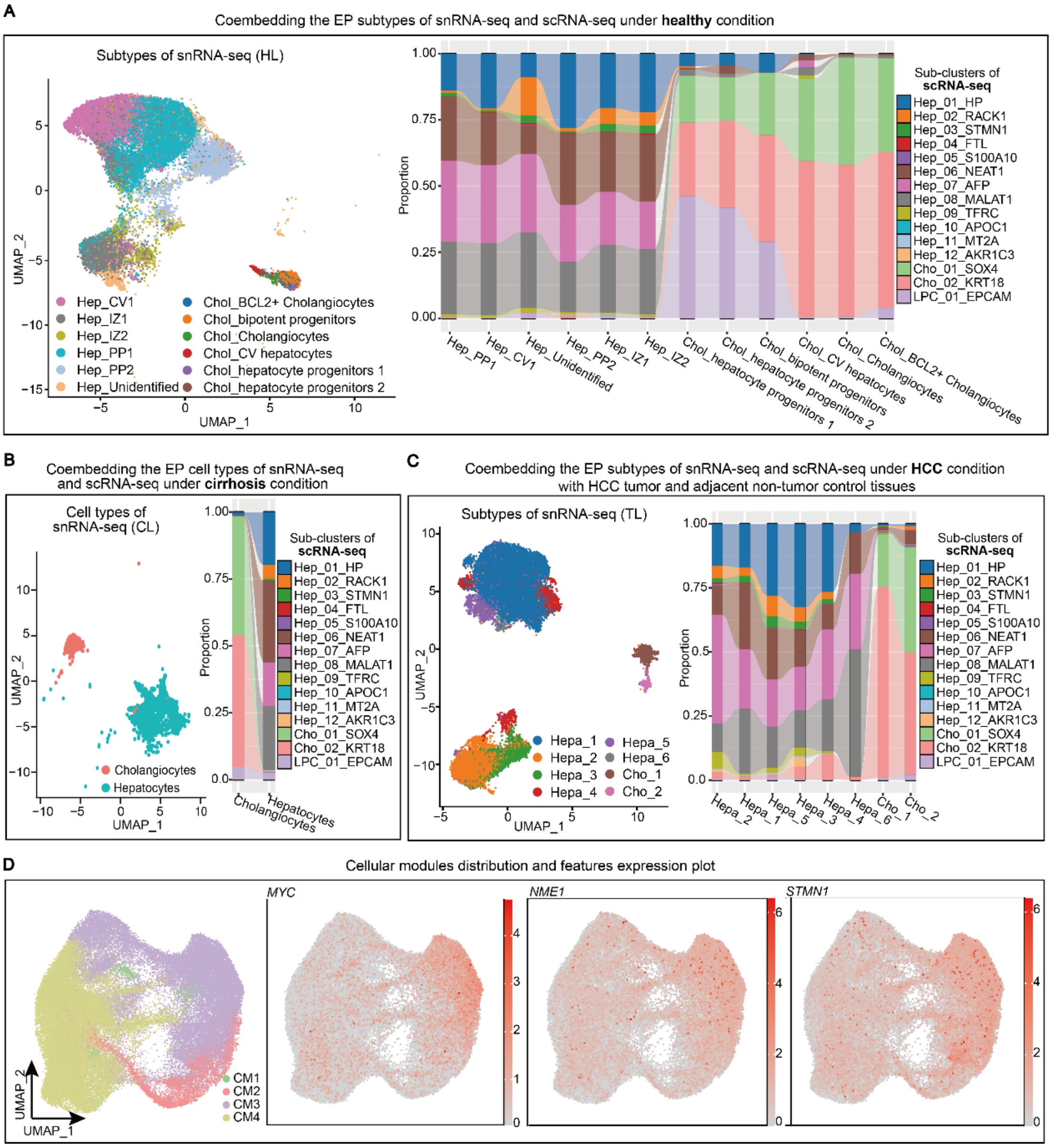

Supplementary Figure 4. Coembedding two platforms' data and cellular module features.

(A) Co-embedding of 6 hepatocyte subtypes and 6 cholangiocyte subtypes from HL snRNA-seq data. (B) Correspondence of hepatocyte and cholangiocyte from CL snRNA-seq data with scRNA-seq sub-clusters. (C) Correspondence of hepatocyte and cholangiocyte subtypes from TL snRNA-seq data with scRNA-seq sub-clusters. (D) UMAP plot of cellular module and genes expression.

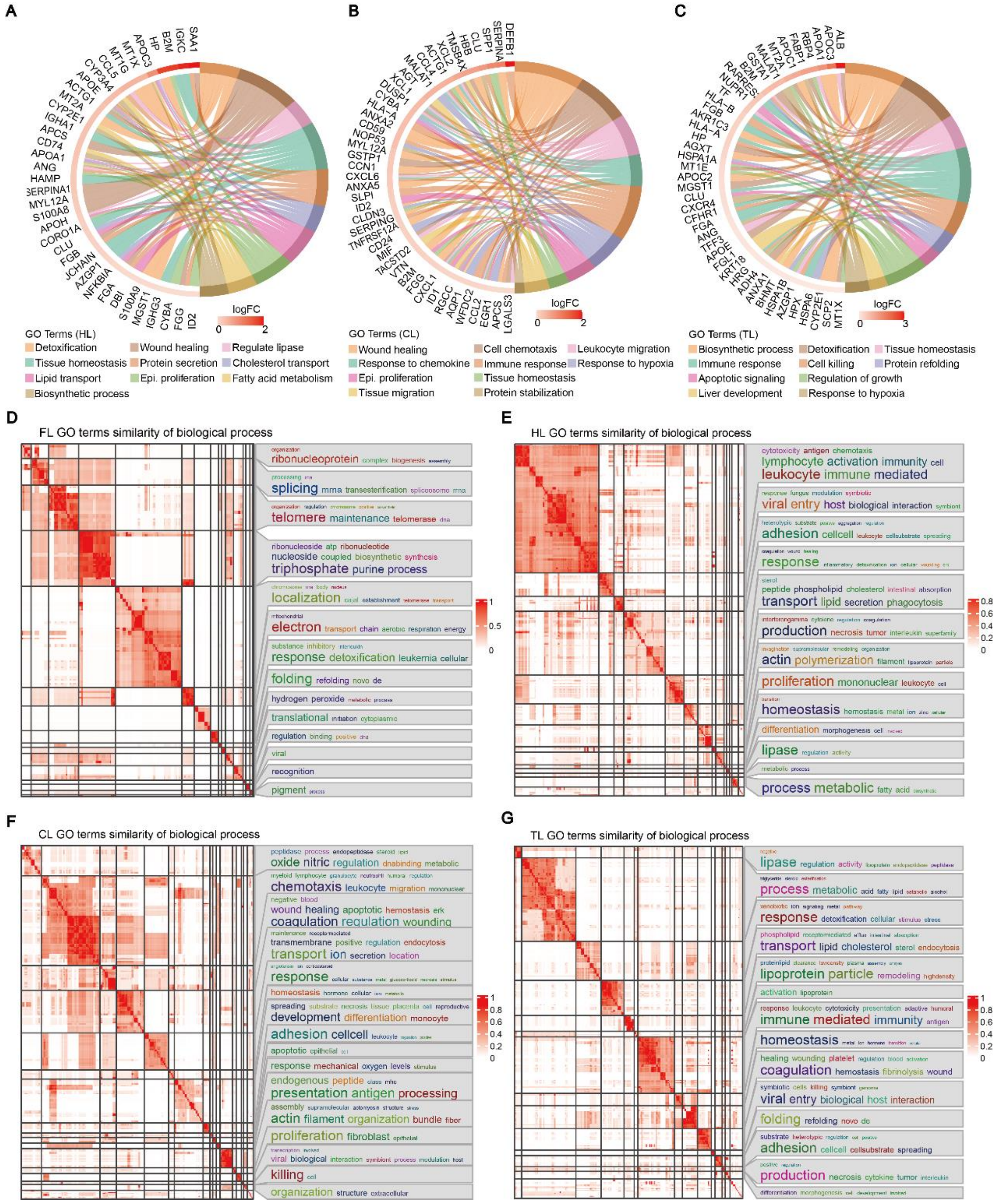

Supplementary Figure 5. Enrichment of Biological Processes in different conditions.

(A-C) The top ten GO terms of biological process in HL, CL, and TL conditions. HL, healthy liver; CL, cirrhotic liver; TL, hepatocellular carcinoma-affected liver. (D-G) GO biological process similarity in four conditions.

EXTENDED METHODS

Human liver tissue

Two human liver tissue samples (STS1, STS2) were obtained from one deceased donor eligible for liver transplantation. The collection was conducted under institutional ethics approval from the Institutional Review Board on Ethics Committee of Beijing Genomics Institute (BGI-IRB 23075). Patient demographic information was gathered and securely stored in an anonymized form. The caudate lobe (segment 1) of the liver was excised during organ preparation for transplantation. Subsequently, two small pieces (50-200 mg each) were extracted from two separate locations of the excised caudate lobe for spatial transcriptome sequencing. The isolated liver blocks were quickly wiped dry with sterile gauze and mixed thoroughly with 4°C OCT (#4583, Sakura). Afterward, the liver blocks were transferred to a metal mold fully embedded with 4°C OCT, quickly frozen on dry ice, and stored in a -80°C refrigerator.

Spatial transcriptome sequencing experiment

We used a standard 1 cm × 1 cm unit of spatial enhanced resolution omics-sequencing (Stereo-seq) RNA capture chip for in situ hybridization as previously described. In brief, these chips contained spots around 220 nm in diameter. They were spaced at a distance of 500 nm between adjacent spot centers, providing up to 400 million spots for capturing tissue RNA per square centimeter. The experimental setup involved precise temperature control (-20°C), pre-cooling instruments in a cryostat chamber, and sectioning an OCT-embedded tissue block in a Thermo Fisher Cryostar NX50 to obtain frozen slices for analyses. The 10-μm thick Stereo-seq slice was carefully transferred onto a -20°C cryostat's cold metal surface. After gently removing scattered tissue debris with a pre-cooled soft brush, the slice was precisely positioned onto the pre-chilled -20°C Stereo-seq chip. Ensuring uniform tissue adhesion, the slice was gradually warmed on the chip, preventing air bubbles and tissue folding. Subsequently, attached tissue slices were incubated with a Stereo-seq chip at 37°C for 3-5 minutes, optimizing experimental conditions. The RNA quality was assessed with a focus on optimizing tissue permeabilization for mRNA release in Stereo-seq experiments. Various durations (6, 12, 18, and 24 minutes) were tested using the STOmics® Stereo-seq Permeabilization Kit. Permeabilization reagent was applied strategically to achieve complete coverage on the chip, incubated at 37°C, and evaluated based on fluorescent image intensity for optimal duration selection. The

experiment involved processing tissue with methanol fixation, fluorescent staining, capturing nuclear ssDNA fluorescent images, and image analysis. After applying a permeabilization reagent and rinsing, in situ reverse transcription and amplification were performed using the Stereo-seq Transcriptomics T Kit. The resulting cDNA underwent fragmentation, PCR amplification, and sequencing on an MGI DNBSEQ-Tx sequencer.

Stereo-seq data processing

Initially, raw reads from Stereo-seq FQSTQ are generated using an MGI DNBSEQ-Tx sequencer. These reads undergo processing via the SAW pipeline (available at https://github.com/BGIResearch/SAW), encompassing mapping, merging, registration, counting, and tissue segmentation sub-pipelines. Quality control filters out reads with low-quality bases or errors introduced during sequencing and PCR. Valid reads are mapped to the GRCh38 reference genome using STAR, followed by gene annotation and quantification based on mapped reads with high mapping quality (MAPQ > 10). The gene expression matrix, incorporating spatial coordinates, is further refined through image registration and tissue recognition. The SAW register sub-pipeline aligns the spatial transcriptome matrix to a unified coordinate system, enhancing accuracy across datasets. Tissue cut tools delineate tissue boundaries based on registration outputs, refining the gene expression matrix to include only relevant tissue regions. At a spatial resolution of approximately 0.5μm, this matrix details gene expression profiles at individual spots (bin1). Image-based single-cell segmentation using StereoCell software also integrates tissue fluorescent images with gene expression matrices.[11]

The cell type annotation of each Stereo-seq cellbin was conducted by R spacexr package v2.0.0 RCTD through deconvolution.[12] The integrated scRNA-seq data and cell type information in this study were exported using the Reference function to generate a reference object. The two core functions create.RCTD and run.RCTD were used to obtain weights that determined the cell type. When calculating the spatial distance between each LPC and other cell types, the distance connecting the centers of the two cells is treated as the hypotenuse, while the horizontal distance in rows and vertical distance in columns are considered the right-angle sides.

Single-cell gene expression matrices collection and gene revision

Gene expression matrices (GEMs) of the fetal liver (FL) with 6 individuals,[6,13] healthy liver (HL) with 14 individuals,[6,13–15] cirrhotic liver (CL) with 5 individuals,[15] and HCC from 17 individuals with 15 tumors (TL) and eight adjacent tissues (TLA),[6,16] consisting of 42 individuals, were collected from Gene Expression Omnibus (GEO) with accession GSE134355,[13] GSE136103,[15] GSE146115,[16] GSE115469,[14] and GSE156337.[6] The gene names from divergent matrices were revised after collecting original GEMs with gene expression counts. Synonymous gene symbols were renamed to official identifiers according to NCBI gene to accession (ftp.ncbi.nih.gov/gene/DATA/gene_info.gz). Finally, we obtained the scRNA-seq dataset with gene name revised matrices.

scRNA-seq matrices quality control

For each scRNA-seq GEM, the data quality control was conducted using the Seurat (v4.3.0),[17] a tool kit within R software. The CreateSeuratObject function of Seurat was used to generate an R object. Low-quality cells and genes were filtered based on four metrics: (1) the genes detected were above 200 and below 6000; (2) the total UMI counts per cell were above 150; (3) the percentage of mitochondrial genes was below 50; (4) genes detected in at least three cells. Next, a Bayesian method of DecontX[18] was used to estimate contamination levels and remove ambient RNA expression in each matrix; cells with a decontXcounts-calculated value exceeding 0.4 were excluded. DoubletFinder was used to identify potential doublets, with a 5% threshold for doublet formation rate.[19]

Data integration and clustering

We conducted a step-by-step integration process to integrate the divergent scRNA-seq data. Normalization and variance stabilization were performed on qualified cells using regularized negative binomial regression with the function SCTransform.[20] Subsequently, 3,000 highly variable genes (HVGs) were selected from all matrices with the SelectIntegrationFeatures function. Then, the top 50 principal components (PCs) were calculated using the RunPCA function. The Harmony algorithm incorporated donor,[21] platform, and sorting strategies as three technical covariates for correction purposes, assigning corresponding theta values to 4, 2, and 2, respectively. We used kBET to evaluate Harmony's batch-regression approach and quantify its effectiveness.[22] The FindNeighbors function was used to identify a shared nearest

neighbor (SNN) graph of the batch-corrected integrated dataset. Subsequently, the dimensional reduction was performed using the RunUMAP function. We then employed the FindClusters function to determine clusters, setting the resolution parameter to 0.8, 1, 2, and 4. Finally, we selected the cluster with a resolution of 2 for cell type assignment.

After cell type assignment, we performed second-round clustering. First, we integrated hepatocytes, cholangiocytes, and LPCs using the canonical correlation analysis (CCA) algorithm to identify integration anchors with the FindIntegrationAnchors function in Seurat. We then applied the IntegrateData function to complete cell integration based on the identified anchor set. To minimize the interference of malignant cells on LPCs, we performed clustering with a series of resolution values (0.2, 0.5, and 0.8). The clustering result with a resolution value of 0.5 was selected, as it provided the most distinct distribution of cell types and DEGs. We identified HCC-specific clusters based on the proportion of each cluster across different experimental conditions and individuals.

Marker identification and cell type assignment

Firstly, the function PrepSCTFindMarkers was used to adjust SCT counts based on minimum median counts among the individuals. Then, cluster-specific markers were determined using the FindAllMarkers function with the Wilcoxon test. Subsequently, major cell types were assigned based on the classic markers of EPs, endothelial cells (ECs), mesenchymal cells, myeloid cells, lymphoid cells, and cells related to erythropoiesis, including hepatocytes (*ALB*, *GLUL*, *TF*),[23] cholangiocytes (*KRT19*, *SOX9*),[24] ECs(*VWF*), hepatic satellite cells (HSCs, *ACTA2*, *LRAT*, *THBS1*),[25] fibroblasts (*TAGLN*, *COL1A1*, *COL1A2*),[26] erythrocytes (*HBB*, *SLC25A37*), erythroblast(*BLVRB*, *PRDX2*), $CD8^+$ T-cells (*CD8A*, *CCL4*, *GZMK*), $CD4^+$ T cells (*CD4*), NK cells (*GNLY*, *GZMB*), monocytes/macrophages (*CD14*, *CD68*, *MARCO*), dendritic cells (*ITGAM*, *ITGAX*, *IL3RA*), B cells (*MS4A1*, *SDC1*, *CD79A*).[27] Based on the traditional progenitor marker *EPCAM*, a progenitor sub-cluster (LPC_01_EPCAM) encompassing 6,754 cells was annotated after second-round clustering.

Coembedding the scRNA-seq and single nucleus RNA sequencing data

In order to validate the representation of EPs identified from scRNA-seq datasets in capturing comprehensive cellular heterogeneity across these conditions, we collected human single-nucleus RNA sequencing (snRNA-seq) data from HL, CL, and TL

conditions to assess the consistency between snRNA-seq and scRNA-seq subpopulations. The following steps were performed for integrating and mapping the snRNA-seq data with scRNA-seq data: 1) Subpopulations of hepatocyte, cholangiocyte, and LPC were extracted from the process files of HL (GSE185477)[28] and CL (GSE202379)[29] conditions. 2) The snRNA-seq matrices of TL condition (GSE189175)[30] underwent quality control and clustering as previously described. 3) Hepatocyte and cholangiocyte subpopulations were then extracted after assigning clusters to cell types using marker genes. 4) The functions FindTransferAnchors and TransferData were utilized to establish the correspondence between cell types in snRNA-seq and scRNA-seq clusters. 5) The proportions of each snRNA-seq sub-cluster mapped to the corresponding scRNA-seq sub-cluster were calculated. Finally, UMAP plots of snRNA-seq sub-clusters and their corresponding composition in scRNA-seq were plotted for each condition to evaluate the presence of corresponding subpopulations between snRNA-seq and scRNA-seq.

Cellular modules of epithelial cells

To determine the potential cellular pattern of LPCs across four conditions, we investigated the co-existence module of EP subpopulations. First, pairwise correlation values between the normalized frequency of any two clusters were calculated using the R cor function. Subsequently, these values were clustered using the corrplot R package with the ward.D cluster method and correlation distance. As a result, four correlated cellular modules (CMs) were identified. Differentially expressed genes (DEGs) among the CMs were then identified, following the procedure described in the previous step. To assess the variation in gene expression patterns across the four CMs, we compared the top 250 DEGs for each CM with the other three CMs. The gene ontology (GO), Kyoto Encyclopedia of Genes and Genomes (KEGG), and gene set enrichment analysis (GSEA) enrichment were performed on DEGs using the R package clusterProfiler.[31] GSEA was performed with hallmark gene sets[32] as input reference terms and corresponding mapped genes.

Module score calculation for regeneration-related genes

To analyze the average expression levels of genes (*EPCAM*, *TACSTD2*, *FGFR2*, *TM4SF4*, *CLDN1*, *ANXA4*, *WWTR1*, *MYC*, *STMN1*, *PSMA4*, *SNRPB*, *ERH*, *NME1*, *TMEM14B*) associated with liver regeneration, we calculated the module score using the function AddModuleScore. The score for each feature was computed with twenty

bins of aggregate expression levels for all analyzed features. One hundred control features were also selected from the same bin.

Cell developmental trajectory of LPCs

We employed the Monocle for LPCs trajectory construction and pseudotime analysis.[33] First, the function GetAssayData was used to extract the SCT assay gene expression matrix, and then the function new_cell_data_set was executed to create a new dataset object. Next, the function Embeddings was used to export Uniform manifold approximation and projection (UMAP) coordinates from the Seurat object and import integrated UMAP coordinates to the newly created object. The learn_graph function was involved in learning the trajectory graph. The cells were then ordered in pseudotime, representing their progress through the developmental program. The trajectory construction and pseudotime analysis among LPCs, cholangiocytes, and hepatocytes were performed using spaTrack (https://github.com/yzf072/spaTrack) under the "single-cell" model. LPC3 served as the starting cluster.

Co-expression network analysis of LPCs

Co-expression network analysis was performed through the R package hdWGCNA.[34] The object of LPCs was built with the SetupForWGCNA function, wherein the option gene_select was set to fraction, selecting genes expressed in >5% of cells. Metacells were constructed using the function MetacellsByGroups, aggregating similar cells from the four distinct conditions via the KNN algorithm. Metacells were grouped by conditions, with the aggregation parameter k set to 20. The SetDatExpr function was then employed to store the transformed expression matrix of LPCs. After obtaining the expression matrix, the TestSoftPowers function was used to assess a series of soft power thresholds. Subsequently, the ConstructNetwork function was executed to build the co-expression network. The UMAP algorithm was applied to embed the hdWGCNA network into a low-dimensional manifold. The positioning of each gene in UMAP space was contingent upon its connectivity with the network's hub genes. Four hub genes from each module were annotated, and each module exhibited distinct characteristics.

Bibliography

1. Zhang B, Li M, Kang Q, et al. Generating single-cell gene expression profiles for high-resolution spatial transcriptomics based on cell boundary images. *GigaByte*. 2024;2024(2):1-13. doi:10.46471/gigabyte.110
2. Cable DM, Murray E, Zou LS, et al. Robust decomposition of cell type mixtures in spatial transcriptomics. *Nat Biotechnol*. 2022;40(4):517-526. doi:10.1038/s41587-021-00830-w
3. Han X, Zhou Z, Fei L, et al. Construction of a human cell landscape at single-cell level. *Nature*. Published online March 25, 2020:1-9. doi:10.1038/s41586-020-2157-4
4. Sharma A, Seow JJW, Dutertre CA, et al. Onco-fetal Reprogramming of Endothelial Cells Drives Immunosuppressive Macrophages in Hepatocellular Carcinoma. *Cell*. 2020;183(2):377-394. doi:10.1016/j.cell.2020.08.040
5. MacParland SA, Liu JC, Ma XZ, et al. Single cell RNA sequencing of human liver reveals distinct intrahepatic macrophage populations. *Nat Commun*. 2018;9(1):1-21. doi:10.1038/s41467-018-06318-7
6. Ramachandran P, Dobie R, Wilson-Kanamori JR, et al. Resolving the fibrotic niche of human liver cirrhosis at single-cell level. *Nature*. 2019;575(7783):512-518. doi:10.1038/s41586-019-1631-3
7. Su X, Zhao L, Shi Y, et al. Clonal evolution in liver cancer at single-cell and single-variant resolution. *Journal of Hematology & Oncology*. 2021;14(1):22. doi:10.1186/s13045-021-01036-y
8. Hao Y, Hao S, Andersen-Nissen E, et al. Integrated analysis of multimodal single-cell data. *Cell*. 2021;184(13):3573-3587. doi:10.1016/j.cell.2021.04.048
9. Yang S, Corbett SE, Koga Y, et al. Decontamination of ambient RNA in single-cell RNA-seq with DecontX. *Genome Biology*. 2020;21(1):57-67. doi:10.1186/s13059-020-1950-6
10. McGinnis CS, Murrow LM, Gartner ZJ. DoubletFinder: Doublet Detection in Single-Cell RNA Sequencing Data Using Artificial Nearest Neighbors. *Cell Syst*. 2019;8(4):329-337. doi:10.1016/j.cels.2019.03.003
11. Choudhary S, Satija R. Comparison and evaluation of statistical error models for scRNA-seq. *Genome Biology*. 2022;23(1):27-37. doi:10.1186/s13059-021-02584-9
12. Korsunsky I, Millard N, Fan J, et al. Fast, sensitive and accurate integration of single-cell data with Harmony. *Nat Methods*. 2019;16(12):1289-1296. doi:10.1038/s41592-019-0619-0
13. Büttner M, Miao Z, Wolf FA, Teichmann SA, Theis FJ. A test metric for assessing single-cell RNA-seq batch correction. *Nat Methods*. 2019;16(1):43-49. doi:10.1038/s41592-018-0254-1
14. Goyak KMO, Laurenzana EM, Omiecinski CJ. Hepatocyte Differentiation. *Methods Mol Biol*. 2010;640:115-138. doi:10.1007/978-1-60761-688-7_6
15. Junge N, Sharma AD, Ott M. About cytokeratin 19 and the drivers of liver regeneration. *Journal of Hepatology*. 2018;68(1):5-7. doi:10.1016/j.jhep.2017.10.003
16. Cogliati B, Yashaswini CN, Wang S, Sia D, Friedman SL. Friend or foe? The elusive role of hepatic stellate cells in liver cancer. *Nat Rev Gastroenterol Hepatol*. 2023;20(10):647-661. doi:10.1038/s41575-023-00821-z
17. Yang W, He H, Wang T, et al. Single-Cell Transcriptomic Analysis Reveals a Hepatic Stellate Cell–Activation Roadmap and Myofibroblast Origin During Liver Fibrosis in Mice. *Hepatology*. 2021;74(5):2774-2790. doi:10.1002/hep.31987

18. Xue R, Zhang Q, Cao Q, et al. Liver tumour immune microenvironment subtypes and neutrophil heterogeneity. *Nature*. 2022;612(7938):141-147. doi:10.1038/s41586-022-05400-x
19. Andrews TS, Atif J, Liu JC, et al. Single-Cell, Single-Nucleus, and Spatial RNA Sequencing of the Human Liver Identifies Cholangiocyte and Mesenchymal Heterogeneity. *Hepatol Commun*. 2022;6(4):821-840. doi:10.1002/hep4.1854
20. Gribben C, Galanakis V, Calderwood A, et al. Acquisition of epithelial plasticity in human chronic liver disease. *Nature*. 2024;630(8015):166-173. doi:10.1038/s41586-024-07465-2
21. Alvarez M, Benhammou JN, Darci-Maher N, et al. Human liver single nucleus and single cell RNA sequencing identify a hepatocellular carcinoma-associated cell-type affecting survival. *Genome Med*. 2022;14(1):50-70. doi:10.1186/s13073-022-01055-5
22. Wu T, Hu E, Xu S, et al. clusterProfiler 4.0: A universal enrichment tool for interpreting omics data. *Innovation (Camb)*. 2021;2(3):1-11. doi:10.1016/j.xinn.2021.100141
23. Liberzon A, Birger C, Thorvaldsdóttir H, Ghandi M, Mesirov JP, Tamayo P. The Molecular Signatures Database (MSigDB) hallmark gene set collection. *Cell Syst*. 2015;1(6):417-425. doi:10.1016/j.cels.2015.12.004
24. Trapnell C, Cacchiarelli D, Grimsby J, et al. The dynamics and regulators of cell fate decisions are revealed by pseudotemporal ordering of single cells. *Nat Biotechnol*. 2014;32(4):381-386. doi:10.1038/nbt.2859
25. Morabito S, Reese F, Rahimzadeh N, Miyoshi E, Swarup V. hdWGCNA identifies co-expression networks in high-dimensional transcriptomics data. *Cell Reports Methods*. 2023;3(6):1-27. doi:10.1016/j.crmeth.2023.100498
